# Carbon-neutral power system enabled e-kerosene production in Brazil in 2050

**DOI:** 10.1038/s41598-023-48559-7

**Published:** 2023-12-04

**Authors:** Ying Deng, Karl-Kiên Cao, Manuel Wetzel, Wenxuan Hu, Patrick Jochem

**Affiliations:** 1 German Aerospace Center (DLR), Institute of Networked Energy Systems, 70563 Stuttgart, Germany; 2 Karlsruhe Institute of Technology (KIT), Institute for Industrial Production (IIP), 76187 Karlsruhe, Germany

**Keywords:** Climate sciences, Hydrogen fuel, Energy grids and networks, Renewable energy, Bioenergy

## Abstract

Rich in renewable resources, extensive acreage, and bioenergy expertise, Brazil, however, has no established strategies for sustainable aviation fuels, particularly e-kerosene. We extend the lens from the often-studied economic feasibility of individual e-kerosene supply chains to a system-wide perspective. Employing energy system analyses, we examine the integration of e-kerosene production into Brazil’s national energy supplies. We introduce PyPSA-Brazil, an open-source energy system optimisation model grounded in public data. This model integrates e-kerosene production and offers granular spatial resolution, enabling federal-level informed decisions on infrastructure locations and enhancing transparency in Brazilian energy supply scenarios. Our findings indicate that incorporating e-kerosene production can bolster system efficiency as Brazil targets a carbon-neutral electricity supply by 2050. The share of e-kerosene in meeting kerosene demand fluctuates between 2.7 and 51.1%, with production costs varying from 113.3 to 227.3 €/MWh. These costs are influenced by factors such as biokerosene costs, carbon pricing, and export aspirations. Our findings are relevant for Brazilian policymakers championing aviation sustainability and offer a framework for other countries envisioning carbon-neutral e-kerosene production and export.

## Introduction

Addressing the urgent need to achieve net-zero $\hbox {CO}_{2}$ emissions by 2050 and net-zero greenhouse gas (GHG) emissions by 2100 is critical for maintaining global warming well below 1.5 $^\circ$C^1,2^. While many sectors contribute to these mitigation efforts, the aviation sector, a significant emitter of GHG emissions, faces challenges in reversing its rising emission trends^3^. Despite other sectors, which can avoid direct GHG emissions by electrification, this option seems impractical for aviation due to low energy density of current batteries^4^.

Among mitigation strategies for aviation, the adoption of sustainable aviation fuels (SAFs) appears to be a promising solution^5,6^. SAFs comprise both biokerosene and e-kerosene^7,8^. E-kerosene is synthesised from atmospheric carbon dioxide ($\hbox {CO}_{2}$) through direct air capture and hydrogen ($\hbox {H}_{2}$) derived through the electrolysis of water using renewable electricity^9^. E-kerosene offers the potential to enable carbon-neutral aviation and may address some of the sustainability concerns associated with the mass production of biokerosene^9–11^.

Regions such as the European Union proposes to make SAFs 63% of aviation fuel by 2050, specifically targeting a minimum of 28% for e-kerosene^12^. Germany, in collaboration with various industry stakeholders, plans to scale e-kerosene production to a 2% share by 2030^13^. Brazil, rich in renewable resources, expansive geography, and long-standing bioenergy expertise, is uniquely positioned to accelerate e-kerosene adoption and become a competitive green energy exporter^8,14^. However, Brazil lags in formulating a national strategy for SAFs^15^. The production costs for biokerosene in Brazil for 2030 are anticipated to range between 58 €$_{2019}/\text{MWh}$ and 197 €$_{2019}/\text{MWh}$^16,17^. Nevertheless, the potential role and contribution of e-kerosene within this context are yet to be delineated.

Affordably producing e-kerosene at scale requires large amounts of cheap renewable electricity, which accounts for about 53% of the total cost^8,13^. With Brazil’s pledge to climate neutrality by 2050^18^ and its highly renewable energy mix^19^, further infrastructural advancements towards non-hydro renewable sources and clarity on system integration across sectors become imperative^20–22^.

High cost and uncertain availability, however, offset the large-scale adoption of e-kerosene in the aviation sector^6,23^. A predominant approach for its economic assessment is the Techno-economic Assessment (TEA), which yields a wide range of e-kerosene production costs, from 69 to 434 €/MWh for 2050^8,24–29^, primarily due to the differences in applied methodology, level of detail, system boundaries, and assumptions about techno-economic factors^30^. An energy system analysis (ESA) approach offers a system-wide perspective and captures the interconnections between the components of a real-world energy system^31^. In addition, unlike TEAs, which often feature static renewable electricity costs (cf. References^23,28,32,33^), ESA models the endogenous costs and supply of renewable electricity while balancing supply and demand^31^, which leads to better accuracy in fuel costing^34^. An optimisation-based TEA integrates system dynamics to consider electricity-related components like electric heating and storage^27^. However, this study only examines specific US locations and neglect the local economic viability of e-kerosene production throughout the country. On the other hand, studies exploring the nationwide generation potential of renewable energy, the focus is often not on e-kerosene specifically (cf. References^35,36^). In particular, most ESA studies contemplate minimal or no use of e-fuels by assuming deep or full direct electrification of one or all end-use sectors (cf. References^37,38^). For those envisioning the use of e-fuels in energy system models, the form of e-fuels does not centre on e-kerosene but rather biofuels^39^, hydrogen^40^, methanol^34^ and methane^34,40^. In those studies, the aviation sector either receives no attention^41^ or is folded into the broader transportation sector^42^. Even studies adopting a system perspective to endogenously calculate electricity prices rarely root the prospect in a local context across the country^42^. As a result, little is currently known about the possibility of simultaneously scaling up e-kerosene and renewable electricity production nationwide in Brazil.

While previous studies analysing green energy exports pinpoint optimal locations for renewable resource harnessing, they often exclude domestic electricity supply and local consumption of energy carriers within the exporting country. For example, studies have considered hydrogen production in Argentina’s Patagonia for export to Japan^43,44^, China’s offshore capabilities to meet Japan’s hydrogen demand^45^, the use of optimal PV and wind resources for methane and diesel production in the Maghreb region, and the production of various e-fuels in Morocco for export to Europe^46,47^. In addition, Hampp et al.^48^ centres exclusively on exporting e-fuels like hydrogen and methane from various countries (i.e., Spain, Denmark, Morocco, Egypt, Saudi Arabia, Argentina, and Australia) to Germany, bypassing domestic usage considerations.

In summary, while e-kerosene presents a potential sustainable solution for the aviation sector, several gaps in its production and integration into the larger energy system remain, especially for Brazil. These gaps include (1) limited research on the potential of e-kerosene production in Brazil, (2) insufficient consideration of e-kerosene from a potential exporter’s perspective, and (3) lack of nationwide assessments of concurrent scaling of e-kerosene and renewable electricity. In light of these observations, our study seeks to provide insights into optimising e-kerosene production in Brazil, particularly from an ESA viewpoint, balancing domestic energy needs and potential export opportunities. We further discuss the trade-offs between e-kerosene supply and competing options such as biokerosene and conventional kerosene. We propose the following research questions to guide our study:

1. Is renewable energy sufficient to meet Brazil’s electricity demands in 2050 if e-kerosene fully replaces conventional kerosene?
2. What could a future carbon-neutral power system look like with and without e-kerosene production?
3. In light of uncertain biokerosene production costs and carbon prices, what might be the share of e-kerosene in Brazil?
4. What could be the export cost if Brazil becomes an exporter of carbon-neutral kerosene?

## Results

The following sections are structured according to the research questions outlined earlier. Further elaboration can be found in Supplementary Section E.1.

### Availability of renewable energy in Brazil for comprehensive e-kerosene production

We evaluate the model assumptions concerning demand and generation potentials within the PyPSA-Brazil model (cf. Deng et al.^49^ and Supplementary Section C), in light of Research Question 1. It particularly focuses on the potential for renewable energy generation and the projected energy demand in Brazil by 2050, with a special emphasis on e-kerosene.

Table 1 compiles the technical potential for renewable energy generation and contrasts it with statistics on electricity generation for the year 2019. The inclusion of 2019 data helps to interlink the scenario results with the current status of renewable energy generation status in Brazil today (cf. Supplementary Section E.2.4). On the other hand, Table 2 reveals how much energy Brazil could need by the year 2050, including electricity, kerosene (converted into TWh), and annual electricity exchanges.

**Table 1:** Renewable electricity generation potential assumed in PyPSA-Brazil.

Technology	Status in 2019 (TWh)^69^	Potential (TWh)^49^
Offshore wind	–	3552.9
Onshore wind	53.4	3114.0
Photovoltaic	5.0	513,669.2
Hydropower	405.6	2.0$^{\text{a}}$
Non-biomass thermal	73.3	–
Biomass thermal	14.5	222.5$^{\text{b}}$
Nuclear	16.1	–
Total	567.9	520,560.6

${}^{\text{a}}$The computation represents the product of the allowed expansion capacity and the Energy to Power ratio, presented in h.

${}^{\text{b}}$The value is the multiplication outcome of the allowed expansion capacity (set at 25.4 GW) and the cumulative hours of the year, which amount to 8760 h.

**Table 2:** Energy demand in 2050 assumed in PyPSA-Brazil.

Demand type	Input scenario	Value (TWh)
Kerosene	ANAC “with mitigation”	157.0
Electricity^49^	$\text {COPPE}_\text {2Deg2030}$	779.4
	$\text {COPPE}_\text {BAU}$	748.1
	$\text {COPPE}_\text {lowBECCS}$	1167.8
	$\text {PNE2050}_\text {ECS}$	885.3
	$\text {PNE2050}_\text {SS}$	620.8
Electricity import/export$^{\text{a}}$	Import/export from neighbouring country	1.87
Maximum total demand$^{\text{b}}$	–	1321.93

${}^{\text{a}}$The value represents electricity import/export from neighbouring countries. Positive values indicate Brazil importing energy, while negative values indicate Brazil exporting energy. The assumption is based on the electricity trade patterns observed in the base year 2019.

${}^{\text{b}}$The value is calculated as the sum of kerosene demand and electricity demand of $\text {COPPE}_\text {lowBECCS}$, subtracting electricity imports/exports.

Our analysis estimates that the potential renewable electricity generation stands at a substantial 520,561 TWh, far surpassing the maximum total demand of 1322 TWh. Given an e-kerosene production efficiency at 0.42 (cf. Supplementary Section B.3 and Table S5), meeting the entire kerosene demand through e-kerosene would theoretically impose an additional burden of 374 TWh. This amount corresponds to 32% of the future electricity demand under the $\text {COPPE}_\text {lowBECCS}$ scenario. Hence, the main obstacle to building adequate infrastructure to harness the generating potentials would principally revolve around economic feasibility and the societal acceptance of renewable power plants.

Acknowledging the restrictions on expanding current hydropower facilities due to environmental and societal considerations^50^, we base our assumption on the growth trajectory laid out in the Brazilian Ten-Year Energy Plan^49,51^. To effectively integrate the production of e-kerosene in the aviation sector into the Brazilian energy mix, strategic decision-making must be made regarding locations and timings for electricity and e-kerosene production, with consideration of the aforementioned constraints and potentials.

### Future carbon-neutral power system with and without e-kerosene production

In response to Research Question 2, we conduct a comparative analysis of Brazil’s future carbon-neutral power system, specifically examining the incorporation of e-kerosene production. Two scenarios are compared based on the distribution of optimal installed capacity and the Averaged System Cost (ASC). We consider capacity distribution to highlight strategic allocation of various energy sources across regions. The ASC, meanwhile, provides a normalised measure of the economic burden of the power system, accounting for both the capital and operational expenditures relative to the energy generated (cf. Supplementary Section D.1). Supplementary Section E.1 further details two other pivotal factors: annual total system costs and electricity generation (national-wise aggregated).

**Figure 1:**
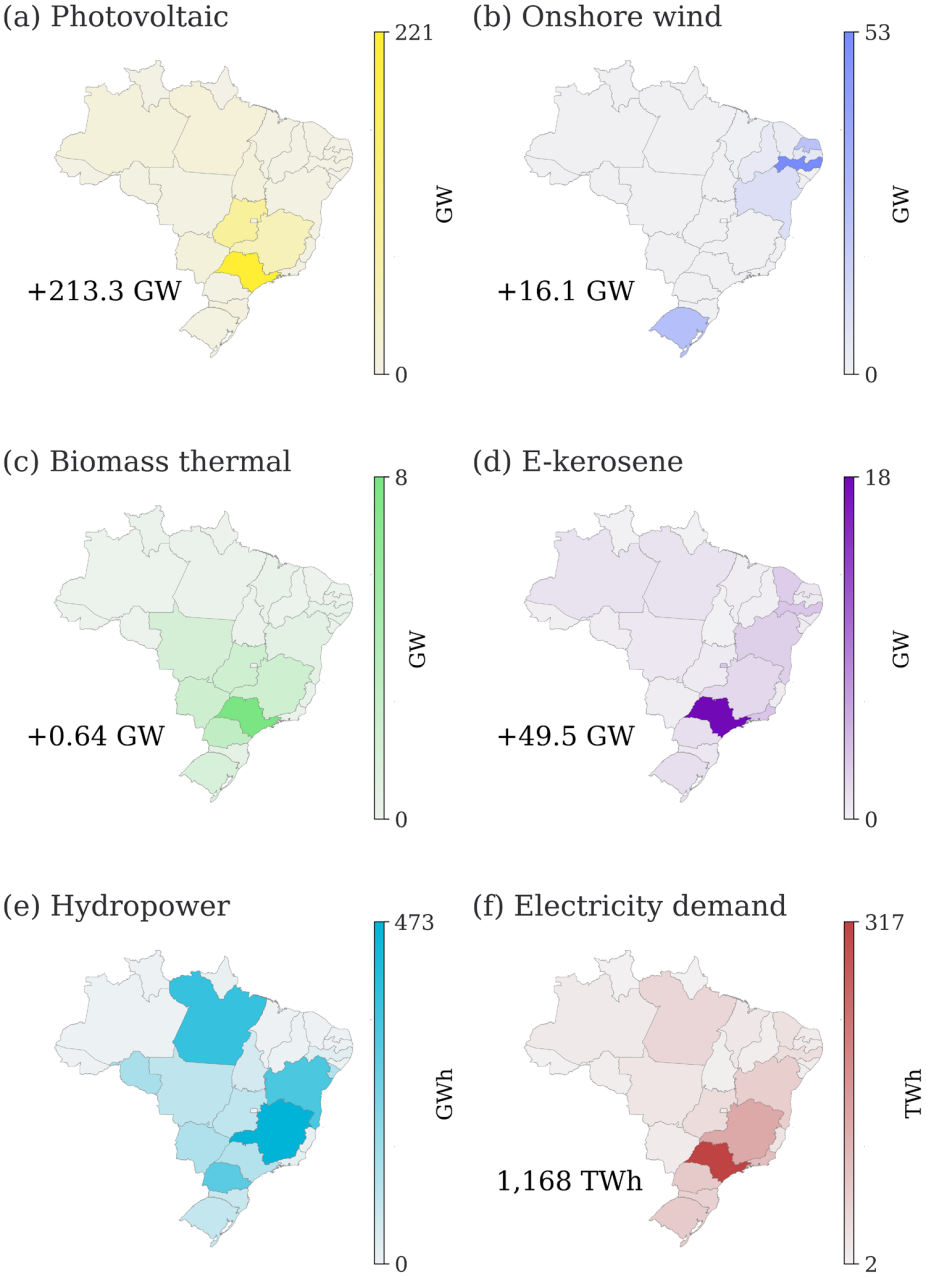
Distribution of installed capacities of selected technologies in 2050 for the “100% e-kerosene supply” scenario obtained from PyPSA-Brazil. The values indicate the differences compared to the “only power system” scenario. Note that, unlike the other subplots, (f) electricity demand is an input to the model and is included here for reference.

The efficient allocation of generation capacities across different energy sources and regions can help in minimising costs and ensuring a reliable energy supply^52^. In this context, considering the regional differences between the two scenarios, PV installations display the most pronounced impact with a surge of 213 GW (cf. Fig. 1). The federal states of São Paulo, Minas Gerais, Distrito Federal and Goiás are expected to be the focal regions for PV installations. In conjunction with PV, the expansion of installed onshore wind power capacity is necessary, especially in the Northeast and South regions of Brazil. The state of Rio de Janeiro shows a prominent increase with a total installed capacity of 16.11 GW. The disparity in biomass thermal plant installations is relatively modest at 0.64 GW. To exclusively fulfil the kerosene demand of both domestic and international airlines refuelling at Brazilian civil airports with e-kerosene by 2050, Brazil would have to install a total of 49.54 GW of e-kerosene production units. São Paulo is expected to host the bulk of these installations. The incorporation of e-kerosene into the energy mix calls for an extra 387 GWh of battery storage and 6601 GWh of kerosene tank capacity compared to the scenario with only a carbon-neutral power system. To realise a fully decarbonised power system, the grid is set to grow beyond its size as of 2019. According to our model, this expansion rises to 29.5% when including e-kerosene production to meet the projected kerosene demand in 2050.

Integrating e-kerosene production is found to be economically beneficial, as it leads to 13.8% reduction in the ASC, from 50.3 €/MWh in the “only power system” scenario to a lower value. This decrease can be attributed to a reduction in the curtailment of biomass thermal power plants (falling from 50.5 to 22.5%). Concurrently, offshore wind power generation also experiences a reduction in curtailment (from 10.8 to 6.3%), as does PV power plants (from 0.9 to 0.3%). The exception is onshore wind, which observes a slight increase of 1%. Therefore, our results indicate that the integration of e-kerosene production contributes to improving the overall efficiency of the power system.

### Share of e-kerosene in Brazil under uncertain biokerosene costs and carbon prices

In this analysis, we assess the cost-effectiveness of e-kerosene, biokerosene, and conventional jet fuel in light of the Research Question 3 by revealing their proportions needed to satisfy the kerosene demand across Brazil’s federal states in 2050.

**Figure 2:**
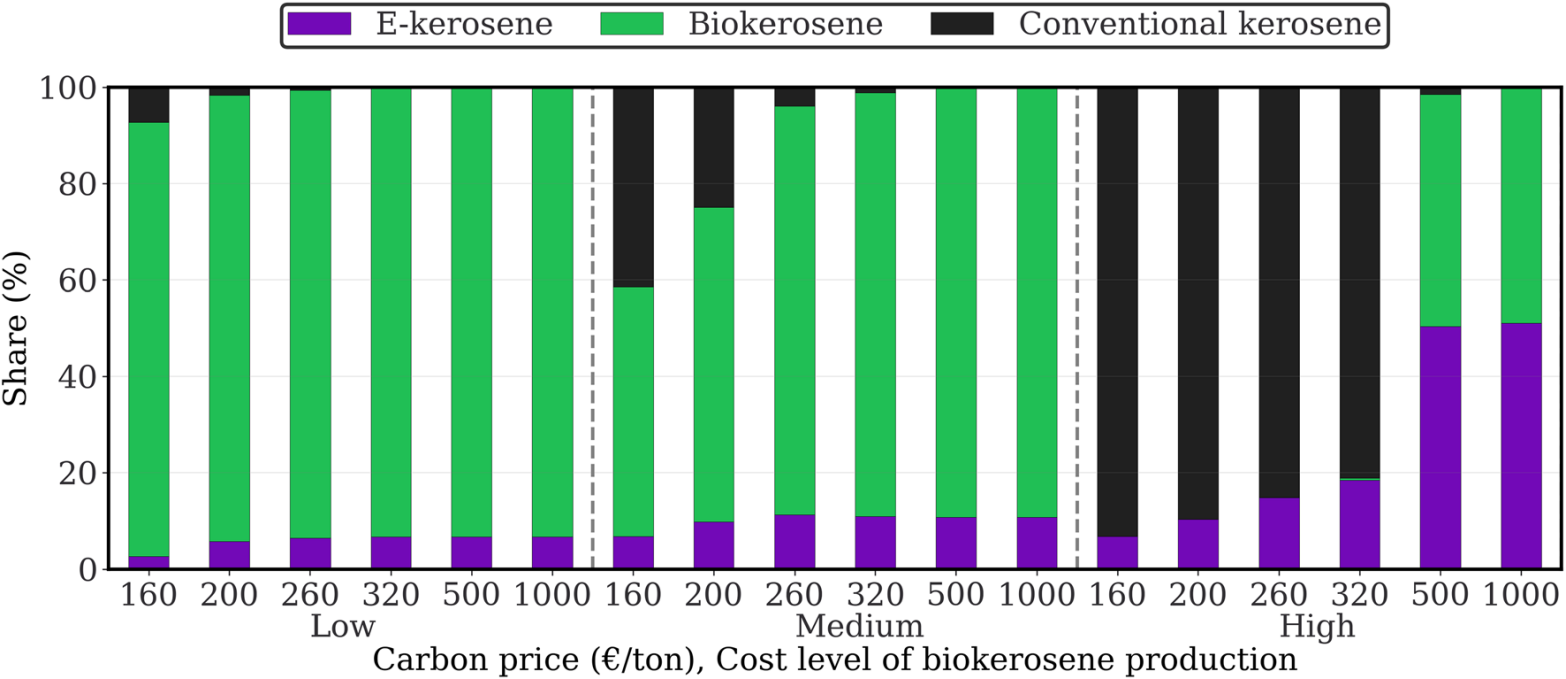
Supply shares of e-kerosene, biokerosene, and conventional kerosene in Brazil, adjusted according to biokerosene production costs and carbon pricing associated with conventional kerosene production in 2050.

According to the model results, e-kerosene constitutes between 2.7 and 18.5% of the fuel mix in most scenarios. These values are derived from the PyPSA-Brazil model under different scenarios of biokerosene production costs and carbon prices (cf. “Methods” section—“Scenarios definition”). In the case where both biokerosene production costs and carbon prices are high, the share of e-kerosene jumps significantly, about 50.4–51.1% of the fuel mix, as shown in Fig. 2. This outcome indicates a positive correlation between the share of e-kerosene and the production costs of biokerosene alongside carbon pricing.

Previous section compares the “100% e-kerosene supply” scenario, wherein the entire kerosene supply is assumed to be e-kerosene, against a carbon-neutral power system scenario (cf. Supplementary Figure S8). We, in Table 3, extends this analysis with further details, contrasting scenarios in which kerosene demand is exclusively met by conventional kerosene at various carbon prices (0 €/t, 500 €/t, and 1000 €/t), or by biokerosene with different production costs (low, medium, and high). Under the assumption that e-kerosene is the only source to meet the kerosene demand by 2050, we find that the total system cost rises by 29.1%. This is only more cost-intensive than scenarios where kerosene demand is completely fulfilled by low-cost biokerosene (22.5%) or carbon-free priced conventional kerosene (19.3%). Therefore, if the conventional kerosene costs remain as they are today and the biokerosene costs stay low in the future, their economic competitiveness may discourage the production of e-kerosene.

**Table 3:** Comparison of additional theoretical costs on a carbon-neutral power system basis when the total domestic kerosene demand in 2050 (157 TWh) is covered by conventional kerosene, biokerosene and e-kerosene$^{\text{a}}$.

Kerosene options	Absolute difference (billion €/year)	Relative difference$^{\textbf{b}}$ (%)
Conventional		
No carbon price	11.4	19.3
Carbon price: 500 €/t	32.3	54.7
Carbon price: 1000 €/t	53.2	90.0
Biokerosene		
Low costs	13.3	22.5
Medium costs	19.3	32.7
High costs	33.3	56.3
“100% e-kerosene supply” scenario	17.2	29.1

${}^{\text{a}}$Results are obtained by post-processing, except for the “100% e-kerosene supply” scenario.

${}^{\text{b}}$The “only power system” scenario projects a system cost of 59.1 billion €/year.

### Export costs of carbon-neutral kerosene from Brazil

Drawing from the cost-benefit analysis in the aforementioned subsection, it is identified that when the carbon price reaches 1000 €/t, only carbon-neutral kerosene—comprising biokerosene and e-kerosene—is integrated into the supply, regardless of how the biokerosene production costs vary (cf. Fig. 2). Building on this finding, this section explores the implications of exporting carbon-neutral kerosene, in response to Research Question 4.

**Figure 3:**
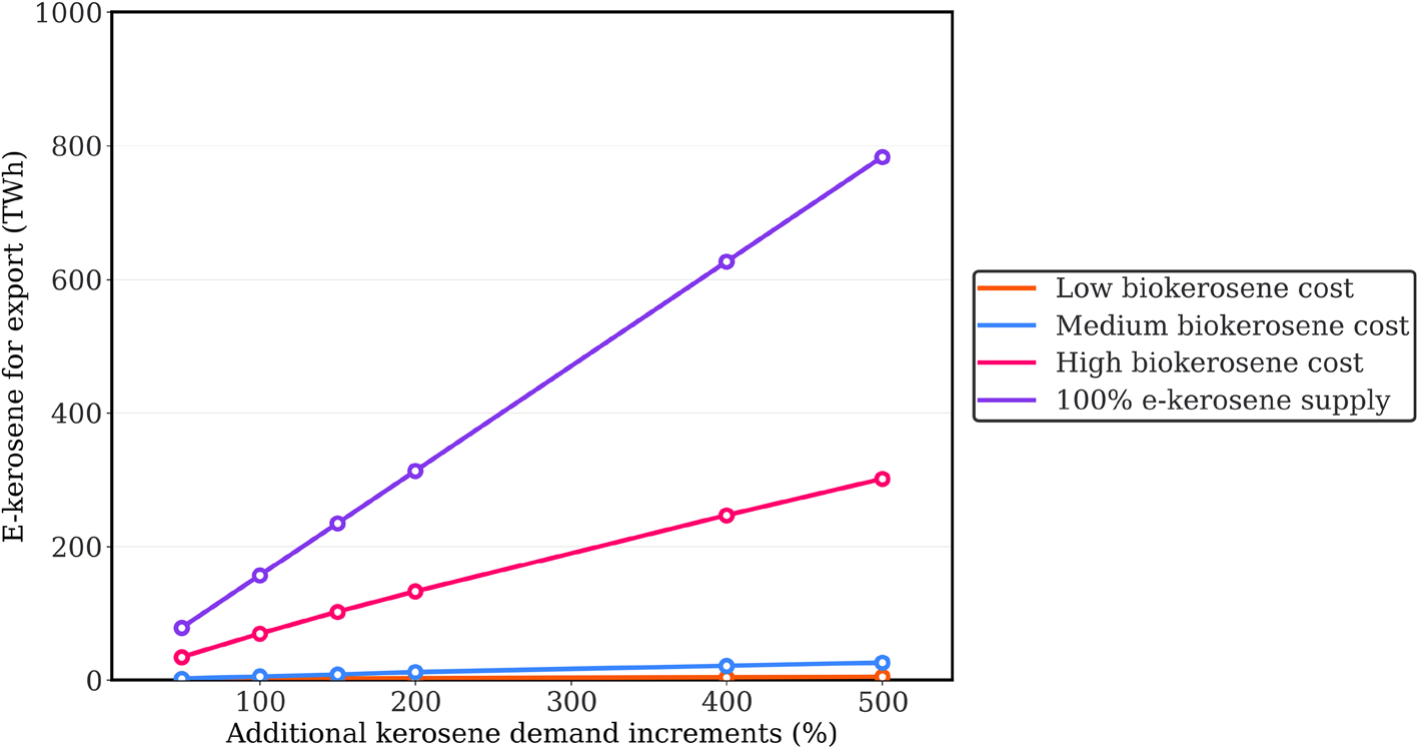
E-kerosene generation for export at different cost levels of biokerosene production, assuming a carbon price of 1000 €/t.

According to our analysis, the export costs of carbon-neutral kerosene (cf. Supplementary Section D.4), tend to remain stable despite increasing export demand. However, these export costs range from 78 to 181 €/MWh, depending on whether the production costs of biokerosene are low or high. It is also noteworthy that the export costs under high biokerosene production costs are approximately equivalent to the cases restricted to e-kerosene production. Figure 3 further elucidates this by presenting the model outcomes of e-kerosene generation for export (cf. Supplementary Section D.5) as an illustrative demonstration of its contribution at various cost levels of biokerosene production. For contextual reference, results from the hypothetical scenario where the supply is solely composed of e-kerosene are also exhibited, concurrent with the rising demand for kerosene export. Our results indicate that e-kerosene production for export remains restrained, stabilising below 25 TWh, when the biokerosene production costs are low to medium. The greater cost competitiveness of biokerosene production, particularly in meeting the additional export demand, accounts for this trend over e-kerosene production.

## Discussion

Based on the model results (cf. “Results” section and Supplementary Section E.1), we discuss these findings in detail in the following sections and further enhance our critical analysis in Supplementary Section E.2.

### Feasible e-kerosene production in Brazil from abundant renewable potential

Our analysis reveals that Brazil’s generation potential of renewable energy substantially exceeds the anticipated electricity demand in 2050 under the $\text {COPPE}_\text {lowBECCS}$ scenario. Combining this potential with the expected efficiency of e-kerosene indicates that the added electricity posed by e-kerosene production could be met in addition to the electricity demand from other sectors (cf. “Results” section—“Availability of renewable energy in Brazil for comprehensive e-kerosene production”). Furthermore, our model shows that integrating e-kerosene production into Brazil’s carbon-neutral power system could lead to a reduction in the curtailment of power generation and more favourable ASC (cf. “Results” section—“Future carbon-neutral power system with and without e-kerosene production”).

Therefore, attaining a fully decarbonised power system with e-kerosene production in Brazil is both feasible and more efficient than merely targeting a carbon-neutral power system by 2050. Our modelling results indicate that such a system necessitates the deployment of new PV capacities, particularly in the federal states of Minas Gerais, Goiás, and Distrito Federal, with a special emphasis on São Paulo (cf. Supplementary Sections E.2.1 and E.2.2 for further discussion).

Owing to Brazil’s substantial solar potential and the relatively low cost of PV installations, they hold the leading position in Brazil’s quest for a carbon-neutral power system. Other cost assumptions, such as those made by Dranka and Ferreira^53^, lead to diverse outcomes. Our estimation of Levelised Cost of Electricity (LCOE) for PV in 2050 is at approximately 33 €/MWh, which is in alignment with findings by IEA^52^ and IRENA^54^. The LCOE of other renewable energy technologies, such as biomass thermal plants and onshore wind power, are observed in the same order of magnitude^54^. An exception to this finding is offshore wind, with LCOE estimated at around 59 €/MWh, which is lower than the anticipated range of 71.6–115.3 €/MWh.

### Consistent system benefits from e-kerosene production

Assuming the implementation of a carbon price in Brazil by 2050, from 160 €/t, our model indicates that e-kerosene remains cost-beneficial in various scenarios. Our analysis, detailed in “Results” section—“Share of e-kerosene in Brazil under uncertain biokerosene costs and carbon prices”, demonstrates that the production cost of e-kerosene can be competitive with biokerosene and carbon-priced fossil jet fuel. Notably, e-kerosene can make up 2.7–51.1% of the aviation sector’s fuel supply (cf. Fig. 2). However, an increase in carbon pricing does not invariably result in a linear increment in the share of e-kerosene.

The future production cost of biokerosene stands as a determinant shaping the contribution of e-kerosene within the Brazilian aviation sector (cf. Supplementary Section E.2.3 for further discussion). When the biokerosene production costs are low to medium, the contribution of e-kerosene is minimal yet present. In those scenarios, the continuous use of conventional jet fuel may also be cost-effective, if carbon pricing is not high enough. The extent of e-kerosene’s contribution to the Brazilian aviation sector markedly intensifies only when biokerosene production costs are high and the use of conventional jet fuel is restricted, such as by well-established carbon price or carbon emissions budgets (Fig. 2 illustrates this trend). This finding needs to be interpreted with caution as it is primarily impacted by assumptions on the production cost of biokerosene. Despite that PyPSA-Brazil considers biokerosene supply as carbon neutral, indirect GHG emissions, land use, and competition with food would prevent it from being available at scale. In addition, Cervi et al.^16^ conclude a wide range of production costs for biokerosene (79.2–384 €$_{2019}/\text{MWh}$), depending on the pathways and biomass types. Given these variations, the potential for our overestimation of biokerosene’s economical feasibility cannot be overlooked, particularly in the context of assumed unlimited supply and season-independent availability (cf. “Limitations” section for an in-depth discussion).

### Supporting e-kerosene production through exporting scenarios in Brazil

The PyPSA-Brazil model delineates a range of export costs from 78 to 181 €/MWh for the prospect of bolstering e-kerosene production within an export context in Brazil (cf. “Results” section—“Export costs of carbon-neutral kerosene from Brazil”). Our results reveal a noteworthy dynamic: based on our modelling, as the production costs of biokerosene rise, e-kerosene begins to take a more prominent role. This relationship is evident because when e-kerosene production exclusively satisfies both domestic and export demands, the export costs are almost identical to those in scenarios of high biokerosene production cost. To place these findings in a broader context, we compare our results with the research by Hampp et al.^48^ on FT fuels export. Our maximum export cost (181 €/MWh) is comparable with those reported by Hampp et al.^48^ for exports from Australia and Spain to Germany, yet exceed those for Argentina, Egypt, Morocco, and Saudi Arabia. It is worth noting that the methodologies and focuses concerning export product in the two studies are different. Hampp et al.^48^ concentrate on $\hbox {H}_{2}$ export demand, assessing the competitive supply between FT fuels and other chemical carriers, such as Power-to-gas-induced $\hbox {H}_{2}$. Such perspective may overestimate the export costs of FT fuels, especially given the lower efficiency involved in producing FT fuels from $\hbox {H}_{2}$ and then converting them back into $\hbox {H}_{2}$.

### Comparisons with related literature

We position Brazil in the context of global studies on the levelised costs for e-kerosene. While direct comparisons in the model are constrained by the specific focus of our study on Brazil, we reference the Levelised Cost of Fuels (LCOFs) for e-kerosene production in other countries from existing literature (cf. Supplementary Table S8). The other two parameters—the electricity supply cost in a fully decarbonised Brazilian power system and the capacity factor of the e-kerosene supply—are discussed in detail in Supplementary Section E.2.4. This comparative analysis is limited to projections for the year 2050 and integrates production chains for carbon-neutral e-kerosene, consistent with the plant design outlined in Supplementary Section B.3. For Brazil, our LCOFs for e-kerosene range from 113.3 to 215.5 €/MWh, accounting for different carbon prices, biokerosene production costs, and Brazil’s self-sufficiency in kerosene. When considering the export demand for kerosene, these values are adjusted to 117.9–227.3 €/MWh.

Batteiger et al.^8^ estimate the LCOF for Germany to be 186.9 €/MWh, while the Spain’s LCOF is slightly lower at 148.2 €/MWh. Drünert et al.^28^ indicate a wider range for Germany, between 188.4 and 284.3 €/MWh. The variability is likely attributable to disparities in methodologies, electricity costs, and assumptions surrounding the e-kerosene production process. The research by Batteiger et al.^8^, for instance, uses an e-kerosene plant configuration similar to the one we employed. However, they assume electricity costs of 43 €/MWh for Germany and 35 €/MWh for Spain. In scenarios where a decarbonised power supply is considered within the European energy system, Schlachtberger et al.^38^ anticipate electricity costs between 64.8 and 84.1 €/MWh, despite a 5.1% contribution from gas power plants. The range in electricity costs is contingent upon either a nine-fold expansion of the grid or its maintenance at 2013 levels, both of which could lead to a sharp rise in the production costs of e-kerosene when ensuring a carbon-neutral power sector. Meanwhile, our LCOFs for Brazil are relatively high compared to the estimates by Agora Energiewende^29^ and Breyer et al.^25^ for North Africa, the US and the EU-27 region, respectively. These studies report values below 100 €/MWh, which can be attributed to assumptions of lower electricity supply costs in spots with higher renewable energy generation potential. Moreover, sourcing electricity from renewable resources at high generation potentials, without grid expenses and accommodating for final kerosene demand, also contribute to these lower values. The LCOF for the US, as calculated by Sherwin^27^, is at 84.8 €/MWh, lower than that of our estimates for Brazil. This discrepancy is primarily due to their consideration of a more flexible and cost-effective e-kerosene production chain, including lower renewable electricity costs and higher efficiency in converting electricity to e-kerosene. Specifically, Sherwin^27^ assumes a renewable electricity costs of 10 €/MWh for solar, 16 €/MWh for wind and 54.6 €/MWh for grid electricity. Those assumptions are more ambitious than our values for Brazil, where our LCOE is 33 €/MWh for PV, 35 €/MWh for onshore wind and 70 €/MWh for grid electricity. Sherwin^27^ also assumes a higher efficiency in converting electricity to e-kerosene at 0.53, while we assume an efficiency of 0.42. Conversely, Becattini et al.^24^ report the highest LCOF range (217.0–434.1 €/MWh) without specifying a particular country. This indicates the upper limit of costs associated with achieving net-zero $\hbox {CO}_{2}$ emissions in e-kerosene production, depending on production processes and electricity expenses. The lower limit of this range (217.0 €/MWh) is marginally above our LCOF (214.7 €/MWh) where carbon-neutral e-kerosene exclusively fulfils the kerosene demand within a fully decarbonised power system.

In summary, the comparisons suggest that LCOFs for e-kerosene production in Brazil in 2050, although competitive, vary significantly depending on several factors. While Spain and Morocco demonstrate slightly lower LCOFs, Germany’s LCOF figures span a range that overlaps with or exceeds Brazil’s. The US, EU-27, and North Africa demonstrate substantially lower LCOFs, indicating greater competitiveness. Local renewable energy potential and electricity costs are key determinants in these observations^23^. With its potential to transition to a fully decarbonised power system, Brazil’s position in the global e-kerosene market is potentially strong, albeit sensitive to international market dynamics and local policy frameworks.

### Limitations

Our study presents several limitations worth considering. For e-kerosene production, we assume full operational flexibility, thereby disregarding constraints such as plant start-up and shutdown times or associated costs^55^. The hypothesis, although advantageous for the overall system, may not be congruent with the technical and economic specifications of the individual plants^56^.

Our study does not detail the selection or specifications of critical components, such as the electrolyser, synthesiser, direct air capture units, and carbon capture and utilisation with various carbon sources^27,57,58^. This exclusion limits the depth of our insights into the feasibility of e-kerosene production at specific sites. However, integrating sector-coupling technologies could refine the material flow modelling in e-kerosene production, which can enhance our understanding related to the flexible operation of power-to-gas and power-to-liquid applications. Additionally, while our study considers the aggregated electricity demand across various sectors, it does not account for the projected demand for other energy carriers like natural gas and liquid fuels. Given that those energy carriers could be substituted by hydrogen or hydrogen-derived fuels, extending our analysis to include the hydrogen demand could improve the understanding of the system implications^52,59^. Therefore, future research could shed light on the complexities of hydrogen generation, storage, and transportation, as well as low-carbon transition in other sectors in the PyPSA-Brazil model.

For biokerosene, our analysis hinges on a production cost study for 2030 that solely considers first-generation biomass feedstocks in Brazil^16^. The subsequent research by Cervi et al.^17^ broadens the feedstock to include second-generation biomass. A comprehensive evaluation of biokerosene potential in Brazil would ideally encompass the second and third-generation biomass. Complicating matters further, the models employed by Cervi et al.^16,17^ are not publicly available, which restricts external validation and hampers further research. The development of an open-source spatio-temporal techno-economic model, capturing all biomass generations and various environmental factors, would therefore be beneficial.

Lastly, our model idealises both e-kerosene and biokerosene as having zero life-cycle carbon emissions. This assumes no $\hbox {CO}_{2}$ loss between capture and binding in e-kerosene^24^ and net-zero life-cycle $\hbox {CO}_{2}$ emissions in biogenic-kind biokerosene due to full offsetting between combustion and carbon sequestration during feedstock growth^60^. This overlooks significant technical challenges, such as the quest for truly carbon-neutral hydrogen in e-kerosene production^26^ and the variance in well-to-tank GHG emissions in biokerosene production (1.4–37.6$\,{}_{\text{g}}\hbox {CO}_{2^{\text {e}}}/\text{MJ}$)^61^. Our model also neglects non-$\hbox {CO}_{2}$ emissions like $\hbox {NO}_{\text{x}}$, which significantly contributes to formation of ozone and contrail-induced cloudiness at high altitudes, resulting in a net warming effect^3^. For a more complete understanding, future research should integrate more realistic emissions data and account for the multiple variables—technological, economic, social, and political—that influence aviation emissions^62,63^.

## Conclusion

We evaluate the feasibility of e-kerosene supply in a prospective carbon-neutral power system for potential domestic use and export. The methodology encompasses a comprehensive energy system model tailored for Brazil, dissecting three aspects: (1) the fulfilment of aviation fuel demand through e-kerosene, (2) the synergies of attaining a fully decarbonised power system whilst producing e-kerosene, and (3) the trade-offs between supplying e-kerosene and alternate options, such as biokerosene and conventional kerosene. For investigating these elements, the research leverages publicly accessible data and the open-source energy system optimisation model, PyPSA-Brazil. This tool proves a beneficial planning instrument across a 27-node network with hourly resolution.

The findings reveal Brazil’s potential to achieve the (theoretic) dual goals by 2050: establishing a carbon-neutral power system and becoming a prime exporter of carbon-neutral kerosene. This vision is bolstered by system designs where PV assumes the lead technology. A comparison with biokerosene and fossil-derived kerosene highlights that e-kerosene becomes a cost-effective solution, especially when carbon pricing schemes are in place and penalises conventional kerosene supply. The significance of this observation becomes more apparent in scenarios where Brazil actively exports carbon-neutral kerosene. Our research highlights a strong interdependence between the biokerosene costs and e-kerosene supply shares. This suggests that should future challenges, such as competition for land, water scarcity, or political sustainability restriction, increase the cost of biokerosene production, e-kerosene could become the more favourable option.

We step away from traditional narratives, offering Brazil’s first quantitative outcomes for e-kerosene production. By enlarging the scope of the best renewable energy sites to cover a fully decarbonised energy system for e-kerosene production, it imparts valuable insights for fostering the production of e-kerosene. Applying the PyPSA-Brazil model and the associated analysis may serve as a blueprint for other countries.

## Methods

### Optimisation model PyPSA-Brazil

Our analysis uses a novel energy system model, PyPSA-Brazil, specifically tailored for the Brazilian energy context. The model employs publicly accessible data sets (explicated in Deng et al.^49^) and adopts Python for Power System Analysis (PyPSA) framework^64^. A comprehensive description of PyPSA-Brazil—including its rationale, framework selection, formulation, and data sources— is available in the Supplementary Sections A to B.

**Figure 4:**
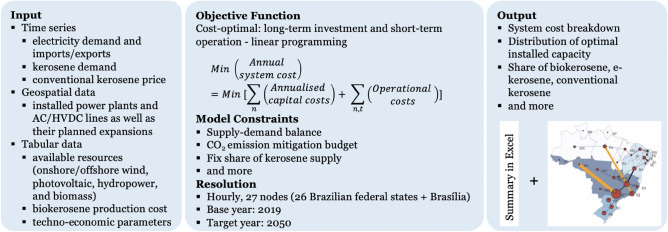
Overview of the PyPSA-Brazil model.

The boundaries of the model are established to ensure the computational feasibility on personal computers, improve accuracy over the existing Brazilian models, and facilitate the investigation of e-kerosene production within the Brazilian power system. As depicted in Fig. 4, PyPSA-Brazil comprises 27 nodes, each symbolising one of the 26 federal states or the federal district of Brasília. Tasked with a cost-optimal equilibrium, PyPSA-Brazil regulates the hourly dispatch and infrastructure expansion across the year to accommodate exogenously designated electricity and kerosene demand, while respecting technical and physical constraints. This objective is set as a linear optimisation problem that factors in short-term dispatch and long-term investments, and is solved using the commercial solver Gurobi^65^.

**Figure 5:**
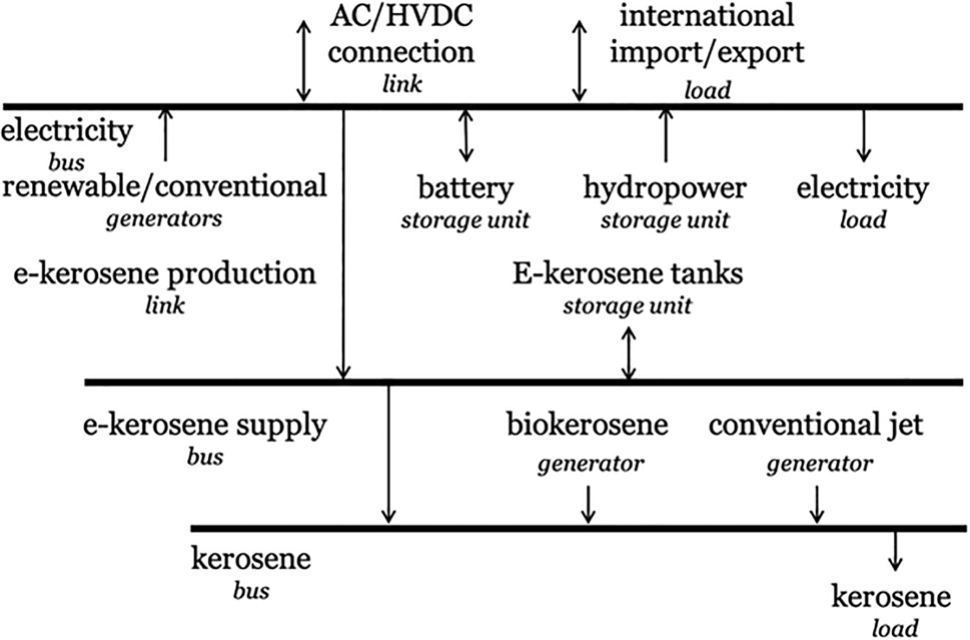
Energy flow at one node in the PyPSA-Brazil model.

The starting point for optimisation is the 2019 power system, inclusive of existing transmission infrastructure, hydroelectric, biomass, wind, and PV facilities, while excluding other existing assets (partial greenfield approach). The capacity expansion of hydropower is assumed, aligning with the National Ten-Year Expansion Plan^51^. See Deng et al.^49^ for details. In accordance with Brazil’s pledge to reach climate neutrality by 2050^18^, our scenario analysis is concentrated on this target year, disregarding the intermediate steps leading up to it. Our model evaluate the potential contribution of e-kerosene production to Brazil’s energy system under the assumption of long-term perfect competition and foresight in market following Neumann et al.^66^.

The parameterisation of the model deliberately avoids favouring certain technology so that the model selects system components solely based on cost-effectiveness and technology characteristics. The Supplementary Section B.3 details assumptions for the e-kerosene production chain, which can vary according to feedstocks and principal technologies^67^.

The application of the PyPSA framework in modelling the technologies in PyPSA-Brazil is depicted in Fig. 5, where energy flow at a single node is illustrated. Italicised terms like Bus, Link, and Generator denote reusable functionality in PyPSA, while arrows represent the flow of energy carriers^68^. Each horizontal line is the bus of energy carriers (i.e., electrical power and kerosene), at which the hourly energy balance should be maintained. Every federal state comprises two buses, representing the power and aviation sectors.

### Scenarios definition

We examine the economic feasibility of the e-kerosene production within a future carbon-neutral Brazilian power system. Additionally, we investigate the prospects of exporting carbon-neutral kerosene from Brazil. The scenarios predominantly align with the Research Questions 1 to 4.

To address the Research Questions 1, we conduct an analytical comparison of electricity demand and generation potential rooted in the input data.

Two scenarios are considered in response to Research Questions 2. The first, “only power system”, posits a carbon-neutral Brazilian power system by 2050 without resorting to fossil and nuclear power plants. The second, “100% e-kerosene supply”, envisages that Brazil completely meets its kerosene demand with e-kerosene by 2050. This spotlights the potential upper boundaries of such a system design.

Research Questions 3 delves into the cost competitiveness of e-kerosene by juxtaposing it with biokerosene and fossil-derived jet fuel. This comparison accounts for all options of kerosene supply, stepping away from the aforementioned ceiling scenario confined to e-kerosene alone. The model favours e-kerosene, biokerosene and fossil-derived jet fuel based on their cost-effectiveness, with the rise in biokerosene production costs and carbon pricing acting as potential catalysts for e-kerosene uptake. The scenario analysis accommodates assumptions on biokerosene inputs (low, medium, and high, cf. Supplementary Section C.4.2). Additionally, it considers varying carbon prices (160 €/t, 200 €/t, 260 €/t, 320 €/t, 500 €/t, and 1000 €/t, refer to Supplementary Section C.6). The model, in return, determines the potential shares of e-kerosene, biokerosene, and conventional kerosene in the total jet fuel demand. By exploring three biokerosene costs scenarios and such a broad range of carbon prices, we aim to understand the sensitivity of e-kerosene’s competitiveness under various carbon pricing scenarios and biokerosene cost scenarios.

For Research Questions 4, we relax the 2050 domestic demand for aviation fuel to consider the potential export expectations. From the findings of Research Questions 3, a carbon price is determined, which encourages the system to supply carbon-neutral alternatives (biokerosene and e-kerosene), irrespective of biokerosene production costs. The model runs through each level of biokerosene production costs with a projected total domestic kerosene demand (i.e., 157 TWh). These runs yield a set of baseline scenarios for subsequent comparative analyses. Following this, the domestic demand is scaled proportionally across Brazil’s federal states with additional increments (50%, 100%, 150%, 200%, 400%, 500%), representing an increased demand for kerosene intended for export.

### Supplementary Information

Supplementary Information.

## Data Availability

We describe the complete data acquisition, modelling, and scenario analysis. Some input data are elaborated upon in Deng et al.^49^. The PyPSA-Brazil code is available on GitLab (https://gitlab.com/dlr-ve/esy/open-brazil-energy-data/pypsa-brazil). The PyPSA-Brazil model is compatible with PyPSA version 0.22.0 and is performed using Python 3.9 and the necessary toolboxes such as Pandas and Geopandas.
