# Author Correction: Carbon-neutral power system enabled e-kerosene production in Brazil in 2050

**DOI:** 10.1038/s41598-024-55392-z

**Published:** 2024-02-28

**Authors:** Ying Deng, Karl-Kiên Cao, Manuel Wetzel, Wenxuan Hu, Patrick Jochem

**Affiliations:** 1 German Aerospace Center (DLR), Institute of Networked Energy Systems, 70563 Stuttgart, Germany; 2 Karlsruhe Institute of Technology (KIT), Institute for Industrial Production (IIP), 76187 Karlsruhe, Germany

Correction to: *Scientific Reports* 10.1038/s41598-023-48559-7, published online 04 December 2023

The original version of this Article contained errors.

In the original version of this article, the names of authors Ying Deng, Karl-Kiên Cao, Manuel Wetzel, Wenxuan Hu and Patrick Jochem were incorrectly given as Deng Ying, Cao Karl‑Kiên, Wetzel Manuel, Hu Wenxuan, and Jochem Patrick.

In addition, the original version of this article contained an error in Figure 5, in which a link arrow was missing. The original Figure 5 and its accompanying Legend appear below.

**Figure 5:**
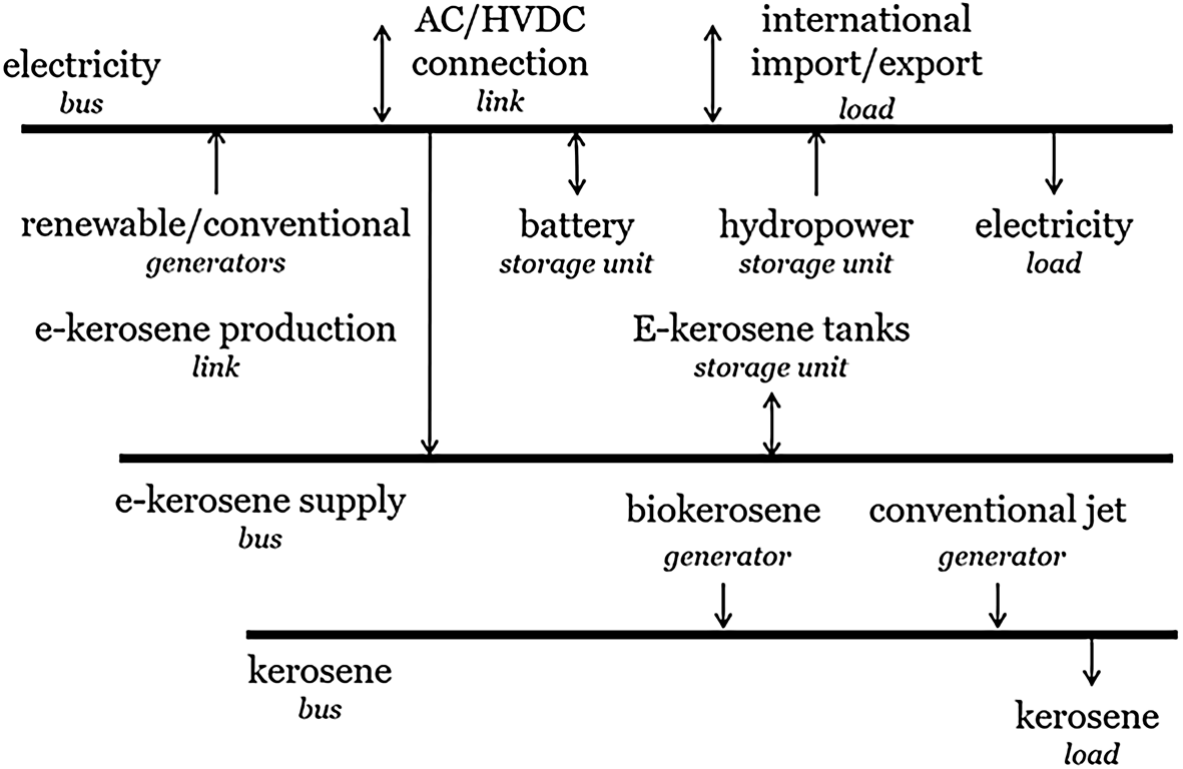
Energy flow at one node in the PyPSA-Brazil model.

The original Article has been corrected.

