# Supplementary material for: Carbon-neutral power system enabled e-kerosene production in Brazil in 2050
Source: Sci Rep. 2023 Dec 4;13:21348. doi: 10.1038/s41598-023-48559-7 (PMC10696038; doi:10.1038/s41598-023-48559-7)
Supplement: Supplementary file 1 — Supplementary Information. [file 41598_2023_48559_MOESM1_ESM.pdf]

Supplementary material

Carbon-neutral power system enabled e-kerosene production in Brazil in 2050

Deng, Ying[1,2,*], Cao, Karl-Kiên[1], Wetzel, Manuel[1], Hu, Wenxuan[1,2], Jochem, Patrick[1,2]

[1]German Aerospace Center (DLR), Institute of Networked Energy Systems, Stuttgart, 70563, Germany
[2]Karlsruhe Institute of Technology (KIT), Institute for Industrial Production (IIP), Karlsruhe, 76187, Germany
[*]dengying8421@gmail.com

A Model framework selection

This section discusses the criteria and processes employed to choose an appropriate modelling framework for our energy system model. We, initially, clarify the need for a novel model in the Brazilian context and describe the model's scope. Subsequently, we elaborate on the methodology used for framework selection.

A.1 Need for a new energy system model for Brazil

A.1.1 High-resolution and open models

Typically, energy system optimisation models are resolved in time, space and on a technological level. Temporal resolutions can be characterised as low (1-12 time slices, e.g., 4 seasons and 3 days/nights), medium (36-288 time slices, e.g., representative days), and high (8,760 time slices, every hour of the year)[S1]. The spatial resolution can be characterised by low spatial resolution – single node modelling – and high spatial resolution for many nodes. The relevant level of techno-economic detail in the model is measured by (i) whether the plant is modelled as fully flexible with fixed efficiency (low level) or as having efficiency delays and time-varying ramp constraints and start-up costs (high level), (ii) whether storage is modelled without (low level) or with self-discharge (high level), and (iii) the lack of generators, storage, and energy demand variability (low level) or presence (high level)[S2].

Models with coarse spatial and temporal resolutions are more solvable and require fewer data[S3]. Nonetheless, applying such models on a national or broader level may engender inaccuracies, especially when wind and solar energy penetration rises[S4]. These inaccuracies stem from the dependence of the economic potential and costs of generating wind, solar and derived energy carriers (such as e-kerosene) on their specific locations and the ability to accommodate fluctuating renewable sources in meeting demand[S5].

Prior Brazilian energy planning studies have employed a few models. MESSAGE-Brazil[S6], BLUES[S7] and REMix-CEM[S8], for instance, are designed for capacity expansion with typical time slices. Noteworthy investigations like Dranka and Ferreira[S9], Gils et al.[S10], and Barbosa et al.[S11], have prioritised time resolution at an hourly scale. These studies, however, limit their scope to predefined transmission among four electric regions, thereby neglecting the benefits and bottlenecks of interstate cooperation. Therefore, Brazil still lacks an energy model that merges both high temporal and federal state-level resolutions.

Furthermore, all of these models that optimise system cost per hour, such as EnergyPLAN[S9], REMix[S10], and LUT Energy System Transition model[S11], are closed source according to Ringkjøb et al.[S3]. This restriction thus challenges more effective and broader collaboration and social debates in practice[S12]. In particular, the lack of open-source models for Brazil complicates understanding the various assumptions made or the interrelationships represented in the mathematical models. As a result, existing model-based scenario analysis may not be fully comprehensible to scientists, stakeholders, and other relevant parties[S13].

A.1.2 Cross-sectoral integration

Future modelling frameworks indicate more cross-sectoral synergy, which helps us understand how various end-use sectors will impact the energy transition[S1]. Several energy studies for Brazil have explored the applications of e-fuels across multiple sectors, such as heating, road and rail transport[S10], seasonal storage for the power sector[S11], and marine shipping[S14]. Yet, a conspicuous shortfall persists – the sector coupling to aviation. Thus, models employed in previous studies must adapt to evolving trends in energy system modelling and the emergence of the aviation sector.

A.1.3 Conclusion

The investigation of sustainable e-kerosene supply in Brazil underscores the crucial role of tailored energy system models. The existing models, as summarised in Table S1, lack the necessary temporal and spatial resolution or are not open-source, making them insufficient for the scope of this research. Thus, it is imperative to conceive a novel energy system model that takes into account the interplay between national power supply and kerosene production. This model should offer a sufficient resolution

for computational solvability, enhance system accuracy under high wind and solar shares, and ensure open accessibility for comprehensive analysis. Our study takes a step in this direction by introducing a new model, as highlighted in Table S1.

Table S1. Comparative overview of energy system model in Brazil: unique contributions of our study.

Study	Model name	Temporal resolution	Spatial resolution	Open source
S6	MESSAGE-Brazil	typical time slices	3 subsystems	No
S7	BLUES	typical time slices	5 regions	No
S8	REMix-CEM	typical time slices	4 regions	No
S9	EnergyPLAN	hourly	4 regions	No
S10	REMix	hourly	13 nodes[a]	No
S11	LUT Energy System Transition model	hourly	5 regions	No
Our study	PyPSA-Brazil	hourly	27 federal states	Yes

A.2 Model scope

As highlighted in Section A.1, the development of a forward-looking open-source model for Brazil's energy system is urgently required to effectively tackle the imperative of decarbonisation in long-term planning. This model of this nature should be capable of regulating load fluctuations associated with the electricity generation from Variable Renewable Energy Source (vRES), while also modelling an array of technologies such as enhanced power transmission, battery storage, or backup generators. Additionally, the model should be flexible enough to include other sectors, specifically addressing the demand and supply of kerosene.

We develop the model with an hourly temporal resolution and a spatial resolution corresponding to the Brazilian federal state. Such a granularity aims to make the model allow for a more detailed and accurate representation of the energy system compared to preceding models, while ensuring compatibility with personal computing devices for user-friendliness.

The high resolution of the model provides policymakers with insights into effective investments in renewable technologies, e-kerosene production units, transmission lines, and storage across federal states. These insights should assist policymakers in avoiding a sharp increase in electricity prices. Thus, the model's objective is cost optimisation of the operation and capacity expansion of the technologies.

A.3 Selection methodology

Energy models typically comprise data and assumptions specific to a particular research purpose. In contrast, energy modelling frameworks consist of architectures designed to provide reusable functionality when building models[S13]. Given the availability of a few energy modelling frameworks, we build on them to create an energy optimisation model for Brazil. A choice of a modelling framework for this study is necessary.

The selection procedure encompasses two steps: preselection and comparison. The information collected is evaluated and assigned a rating according to the satisfaction of the criteria: +2 for fully satisfied, +1 for partially satisfied, and 0 for rarely satisfied (cf. Table S4).

A.3.1 Preselection of modelling frameworks

Preselection criteria include open-source availability, high resolution in time and space, and the capability to analyse multiple energy sectors beyond the electricity sector[S1,S2]. To this end, the selection has been narrowed in accordance with a study of 75 state-of-the-art models and modelling frameworks[S3]. The five preselected open-source modelling frameworks are Calliope, Oemof, PyPSA, OseMOSYS, and FINE (cf. Table S2). These frameworks allow building Linear Programming or Mixed-integer Linear Programming (MILP) models with medium to high temporal and spatial resolution (multi-node approaches possible) while supporting the analysis of sectoral synergies and interconnections.

Table S2. Five preselected open-source modelling frameworks based on the 75 state-of-the-art models[S3, a].

Model	Resolution		Sector-coupling		Model logic		
	in time	in space	commodity	demand sectors	language	methodology	purpose
Calliope[b]	UD[c]	UD	Electricity, heat, hydrogen, fuels	All sectors[d]	Python	LP[e]	Investment & Operation Decision Support
Oemof (SOLPH)[f]	UD	UD	Electricity, heat, hydrogen, fuel	Building, transport, industry	Python	LP, MILP	Investment & Operation Decision Support, Scenario
PyPSA[g]	Hourly	UD	Any commodity	All sectors	Python	LP	Investment & Operation Decision Support, Power System Analysis Tool
OseMOSYS[h]	UD	UD	Electricity[i]	All sectors	GNU MathProg[j]	LP	Investment Decision Support
FINE[k]	UD	UD	Any commodity	All sectors	Python	MILP	Investment & Operation Decision Support

[a] Even though FINE is not featured in Ringkjøb et al.[S3], we collect the information regarding it. Some of this information is updated based on the official documentation of the modelling framework.
[b] Calliope is a model framework published by ETH Zürich (https://www.callio.pe/).
[c] "UD" refers to User-defined. Developers can use the framework to build energy systems with multiple regions (also known as nodes) and time steps at their request.
[d] "All sectors" indicates the common practice in the energy system model of combining demand/load based on power consumption across all sectors.
[e] MILP is under development.
[f] The **Open Energy Modelling Framework**, or Oemof, is an important element of this research published by Reiner Lemoine Institute/ZNES (https://oemof.readthedocs.io/en/latest/).
[g] PyPSA stands for **Py**thon for **P**ower **S**ystem **A**nalysis, published by FIAS[S15] (https://pypsa.org/).
[h] OseMOSYS stands for **O**pen **S**ource **E**nergy **Mo**delling **Sys**tem, published by KTH Royal Institute of Technology (http://www.osemosys.org/).
[i] While only "Electricity" is recognised in [S3, Table 3], the OseMOSYS online documentation indicates its application in the "Water-Food Nexus" project, suggesting the possibility of incorporating multiple commodities within OseMOSYS.
[j] GNU MathProg is a mathematical programming language for describing linear mathematical programming models. In addition, the OseMOSYS model framework is implemented using the languages like GAMS and Pyomo.
[k] The **F**ramework for **In**tegrated **E**nergy System Assessment, or FINE, is published by Forschungszentrum Jülich GmbH (https://vsa-fine.readthedocs.io/en/latest/index.html).

A.3.2 Framework comparison

The preselected frameworks are further compared based on model logic, techno-economic details, ease of use, popularity, and added value.

Model logic evaluations focus on the purpose and language of the frameworks that are compatible with our objective. Except for OseMOSYS, the other four frameworks meet the requirements for cost optimisation for operation and expansion (cf. "purpose" under "Model logic" in Table S2).

Modelling frameworks can be scripted in mathematical programming languages (e.g., GAMS, GNU MathProg, and Pyomo) or general-purpose programming languages (e.g., Python, Julia). While mathematical programming languages offer a closer resemblance to the mathematical model, general-purpose languages are more accessible to non-programmers[S13]. The latter also facilitates data processing and analysis through packages such as Pandas, Numpy, and Matplotlib. For this reason, in the "language" column under "Model logic" in Table S2, a framework based on a general-purpose language is considered more beneficial.

Techno-economic details are essential for the application of modelling frameworks to create energy system models. In modelling the impact of increasing the share of vRES, large-scale production of e-kerosene, and alternate kerosene supply options, key attributes to consider include grid development, energy storage, and demand-side management[S3]. The modeller connects general functions (often called components) by specification and adds user-defined mathematical constraints where necessary. These attributes are the technical and economic parameters in the model components, including conventional or renewable generation technologies, energy storage, emissions, cost, and grid, which determine the level of technical-economic detail. A framework allowing for varying levels of techno-economic details is preferred for flexibility in balancing computational

resources and the consequences of errors due to low resolution of techno-economic detail[S1,S2]. While most of the available model components are similar across the five frameworks, it is the grid modelling that is noteworthy (cf. "Techno-economic details" in Table S3). OseMOSYS does not include grid modelling, Calliope and Oemof (SOLPH) use the Net Transfer Capacity (NTC) approach, while FINE builds on this supports linear power flows, as do power system analysis tools, and PyPSA further allows nonlinear power flows. Thus, PyPSA and FINE are preferred for their relatively great technical-economic details compared to other frameworks.

"Ease of use" is gauged by the availability of comprehensive documents and tutorials that enable entry-level modellers to easily understand the functionality and usefulness of the framework effectively[S13]. From this perspective, the quality and understanding of the online documentation, formulations, and tutorials are characterised as high, medium, or low based on our practical experience (summarised in Table S3 under the "Easy of use").

Up next, the popularity of the framework indicates its sustainability and potential for widespread adoption (cf. "Popularity" in Table S3). The popularity is measured by a combination of factors, including the number of projects, publications, and the presence of an open community. Building a model on a popular framework can enhance its maintenance and likelihood of adoption by other researchers. Choosing a framework with an open community is also beneficial for modellers as it allows for peer-to-peer support and direct discourse with the core developers of the framework[S13]. Evaluating the number of publications, along with the geographic distribution of the most prolific contributors, aids in assessing the international dissemination of the framework.

"Added-value" in Table S3 refers to additional algorithms or embedded libraries within the framework, which relieve modellers of the burden of creating specialised functionalities, such as space and time aggregation. Apart from OseMOSYS, other frameworks provide added value to their frameworks by making extra efforts on additional algorithms or packages.

Table S3. Comparison between the five preselected open-source modelling frameworks[a].

Model	Techno-economic details[b] (generation, storage, grid)	Ease of use[c] (docs, formulation, tutorial)	Popularity[d] projects[f]	publications[g]	open community	Added-value[e]
Calliope	(all[h], all[i], NTC[j])	(high, high[k], high)	EU, UK, Kenya, China, Italy, Bangalore, South Africa, Cambridge	39 (2019-2022), mainly EU + India	Gitter, 66 members	time clustering
Oemof (SOLPH)	(all, all, NTC)	(high, medium, low)	—	95 (2016-2022), mainly EU + China	—	`demandlib` for demand data, `feedinlib` for time series, and `TESPy` provides thermal energy systems for in-depth modelling
PyPSA	(all, all, non-linear/linear power flow[l] and NTC)	(high, medium, high)	EU, South Africa, China, Germany	148 (2017-2022), EU+US	Google group, 388 members	spatial clustering, `Atlite` for vRES generation potential
OseMOSYS	(all, all, None)	(medium, medium, low)	South America, EU, Global, Africa, Cypriot[m]	301 (2002-2022), US+EU	Google group, 381 members[n]	—
FINE	(all, all, linear power flow and NTC)	(low, medium, low)	—	73 (2018-2021), EU+China+US	—	`tsam` for time clustering

[a] The gathered information relies on examining framework documentation, relevant publications, and web sources, as outlined in the associated footnotes.
[b] The provided details are sourced from the[S3] and FINE's documentation. While there is more additional beyond what is presented in Table S2, one can refer to[S3].
[c] The evaluation is conducted by examining the project's source code and documentation.
[d] The data collection phase lasted until October 2021.
[e] Algorithms available to modellers, aiding in workload reduction, are taken into consideration.
[f] The value presented reflects the number of open projects available.
[g] The number is identified through the citation of publications from the Scopus database, which are recommended in the online documentation.
[h] The "all" indicates that power generation technologies, whether conventional or renewable, can be modelled using the provided general functionality.
[i] The "all" signifies that all energy storage technologies, such as batteries, hydrogen, and thermal energy storage, can be modelled with the general functions provided.
[j] The NTC is a popular approach used in energy system models due to its simplicity and high accuracy.
[k] Calliope's clear explanation of its general functionality is particularly useful for developers in technology modelling.
[l] Power flow modelling proves more capable than the NTC approach in grid modelling, as it adheres closely to grid principles. Offering both power flow and NTC approaches is seen as a model selection advantage, providing modellers with more options[S15].
[m] The web documentation indicates the reference to more than five countries.
[n] Of note is the extensive use of OseMOSYS by a great academic community for teaching purposes.

A.4 Selection outcome

Depending on the information collected and shown in Table S2 and Table S3, each framework is scored according to its level of satisfaction with the underlying research criteria. Consequently, PyPSA[S15] emerges as the chosen framework due to its highest score (cf. Table S4).

Table S4. Results of the model frame selection. The orange cell is the preselection step (cf. Table S2), the green cell is the second step of comparison (cf. Table S3), and the obtained scores are listed in the "Total" column. The rating is given according to the level of satisfaction of the criteria, +2 for satisfied, +1 for partially, and 0 for rarely. As shown in the blue cells, PyPSA is the selected model framework.

Open source model	High resolution	Sector-coupling	Model logic	Techno-economic details	Subjective perception	Popularity	Added-value	Total
Calliope	+2	+2	+2	+1	+2	+1	+1	11
Oemof	+2	+2	+2	+1	+1	0	+1	9
PyPSA	+2	+2	+2	+2	+2	+2	+2	14
OseMOSYS	+2	+1	0	0	+1	+2	0	6
FINE	+2	+2	+2	+2	0	0	+1	9

B Model

B.1 Optimisation problem

The objective is to minimise the annual system costs. This includes the annualised capital costs – attributed to the expansion of generation capacities, transmission capacities, storage capacities, and energy conversion capacities – and the variable costs associated with the dispatch of generation, storage, and energy conversion. Equation (1) presents the mathematical expression for this objective:

$$\min_{G,E,F,P,g,e,f,p} \Bigg\{ \sum_{n,r} c_{n,r} \cdot G_{n,r} + \sum_{n,s} c_{n,s} \cdot E_{n,s} + \sum_{l} c_l \cdot F_l + \sum_{n,k} c_{n,k} \cdot P_{n,k} + \sum_{n,r,t} o_{n,r} \cdot g_{n,r,t} + \sum_{n,s,t} o_{n,s} \cdot e_{n,s,t} + \sum_{l,t} o_l \cdot f_{l,t} + \sum_{n,k,t} o_{n,k} \cdot p_{n,k,t} \Bigg\} \tag{1}$$

where c_* represents the capital costs, while o_* signifies variable costs. The indices r, s, l, and k label the generation technologies, storage technologies, transmission lines, and energy conversion technologies, respectively. The symbols G, F, E, and P correspond to the expanded capacity of generation, transmission, storage, and energy conversion technologies, respectively. Additionally, g, f, e, and p indicate the dispatch of the respective elements across various time snapshots, denoted as t and expressed in hours. The index n stands for each node within the system with $n \in \mathscr{N}$, $\mathscr{N} = 1, 2, \ldots, 27$. Transmission lines serve as bus connectors, while specific nodes are equipped with converters that are capable of converting one energy carrier into another. Both transmission lines and converters are modelled using `Link` component, as illustrated in Main Text Figure 5.

The cost of the entire system is derived by summing the annualised capital expenditures, encompassing the annuity payment required and fixed operating expenses, in addition to variable costs, which include variable operating expenses and fuel costs. The equivalent annuity payment required is ascertained by employing the capital investment and the capital recovery factor, in alignment with the method demonstrated by Hörsch et al.[S16], which follows the formula stipulated in Short et al.[S17], taking into consideration the lifetime and discount rate of each technology.

$$\mathscr{A} = \frac{\mathscr{I}}{\frac{(1+i)^N - 1}{i \cdot (1+i)^N}} \tag{2}$$

where

$\mathscr{A}$: equivalent annuity payment
$\mathscr{I}$: capital investment
i : annual discount rate
N : lifetime of the technology in years.

Modelling the physical process entails adopting linear eligibility as a standard assumption.

B.2 Constraints

The system cost minimisation is subject to a set of applied constraints.

B.2.1 Energy balance

The fundamental constraint for optimisation is ensuring that the federal state's energy supply covers the local demand – power and kerosene – for every hour of the year.

$$\sum_r g_{n,r,t} + \sum_s e_{n,s,t} + \sum_l \alpha_{n,l} \cdot f_{l,t} + \sum_k \alpha_{n,k} \cdot p_{n,k,t} = d_{n,t} \quad \leftrightarrow \quad \lambda_{n,t} \quad \forall\, n,t \tag{3}$$

where

$g_{n,r,t}$: generation dispatch
$e_{n,s,t}$: storage dispatch
$\alpha_{n,*}$: flow direction and efficiency on bus connectors – time-independent values – $\alpha_{n,l}$ for transmission lines and $\alpha_{n,k}$ for the production of e-kerosene
$f_{l,t}$: power flow
$p_{n,k,t}$: the dispatch of energy conversion
$d_{n,t}$: hourly demand at node n, either electricity ($d^{\text{electricity}}_{n,t}$) or kerosene ($d^{\text{kerosene}}_{n,t}$)
$\lambda_{n,t}$: Karush-Kuhn-Tucker (KKT) multipliers associated with the equality constraints of the supply-demand balance. The value of $\lambda_{n,t}$ at the optimal point is an output of the optimisation.

The KKT multiplier represents the marginal price of the respective energy carrier at which the node n fulfils more demand at time t, also known as the local marginal price[S18].

The model's hourly resolution significantly lengthens the computation time needed, but it allows for a more accurate system description that considers the synergy effects of various system components or sectors.

B.2.2 Transmission

Between the nodes, the energy can be transferred from one to another through transmission lines. The maximum power flowing through the links is limited by the maximum physical capacity F_l at any time:

$$\underline{f}_l \cdot F_l \leqslant f_{l,t} \leqslant \overline{f}_l \cdot F_l \quad \forall\, l,t \tag{4}$$

where the $\underline{f}_l = -0.7$ and $\overline{f}_l = 0.7$ denote an additional unit security margin for line capacity. These values, on the one hand, secure the approximate N-1 security and reserve capacity for lines, and on the other hand allow both import and export between the neighbouring nodes[S16, S18].

The physical capacity of the line F_l is constrained ($\underline{F}_l \leqslant F_l \leqslant \overline{F}_l$) and can be expanded during the optimisation, depending on the cost-effectiveness. In this study, the lower bound $\underline{F}_l$ is the nominal transfer capacity of lines obtained from Deng et al.[S19]. The upper bound $\overline{F}_l$ is set to infinite. However, the expansion of the transmission lines is limited by a global constraint:

$$\sum_l \ell_l \cdot F_l \leqslant \Gamma_{\text{line volume}} \quad \leftrightarrow \quad \mu_{\text{line volume}} \tag{5}$$

where $\Gamma_{\text{line volume}}$ is the sum of transfer capacities F_l of the line l multiplied by their lengths ℓ_l (referred as line volume and measured in MWkm withing the model) for existing lines with a variable multiplier. Should Equation (5) be binding, the KKT multiplier $\mu_{\text{line volume}}$ is expected to be positive, which represents the marginal value of the increase in line volume to the system. Otherwise, it indicates the cost per MWkm essential for the optimal solution to have the line volume $\Gamma_{\text{line volume}}$[S18]. For the analysis at hand, the multiplier is set to an infinite value, thus making $\Gamma_{\text{line volume}}$ infinite as well.

Linear optimal power flow is applied using Kirchhoff's formulation, which ignores the effect of impedance on the flows and solely requires the nodal power balance according to Kirchhoff's Current Law (KCL)[S20].

B.2.3 Generation

The dispatch of the generators for every hour $g_{n,r,t}$ is constrained by:

$$\underline{g}_{n,r,t} \cdot G_{n,r} \leqslant g_{n,r,t} \leqslant \overline{g}_{n,r,t} \cdot G_{n,r} \quad \forall\, n,r,t \tag{6}$$

where

- $G_{n,r}$: optimum installed capacity of generators
- $\underline{g}_{n,r,t}$: lower bound of the availability. For all types of generators, this value is set to zero ($\underline{g}_{n,r,t} = 0$), which indicates that must-run operation is not required.
- $\overline{g}_{n,r,t}$: upper bound of the availability, [0, 1]. For wind and solar energy, the value is time- and location-dependent. This value refers to its availability per unit capacity. The multiplication value ($\overline{g}_{n,r,t} \cdot G_{n,r}$) indicates the maximum producible energy per hour, which can be derived from the reanalysis weather data. In the context of fossil-fuelled power plants, a default value of 1 signifies high flexibility, devoid of any ramp-up, ramp-down, start-up or shutdown costs.

The installed capacity for generators can be expanded and is limited by the following:

$$\underline{G}_{n,r} \leqslant G_{n,r} \leqslant \overline{G}_{n,r} \quad \forall\, n,r \tag{7}$$

where

- $\underline{G}_{n,r}$: lower bound of capacity expansion. For onshore wind, PV, and biomass thermal power plants, the value is set to be the installed capacity of the base year 2019 provided by Deng et al.[S19].
- $\overline{G}_{n,r}$: upper bound of capacity expansion. The value for wind and solar energy is the technical generation potential estimated by geometric and environmental constraints obtained from Deng et al.[S19]. For biomass thermal power plants, it is the sum of existing and planned capacity for the base year 2019 and the economic potentials presented in[S19].

The PyPSA-Brazil model does not permit the expansion of fossil power plants, and the calculated capital cost is set to zero. However, fossil and nuclear power plants are excluded from our scenario analysis.

B.2.4 Storage

The state-of-charge of the storage should equal the dispatch at each hour:

$$soc_{n,s,t} = \eta_{s,0} \cdot soc_{n,s,t-1} + e^{\text{inflow}}_{n,s,t} + \eta^{\text{charge}}_{s} \cdot e^{\text{charge}}_{n,s,t} - \frac{e^{\text{discharge}}_{n,s,t}}{\eta^{\text{discharge}}_{s}} \quad \forall\, n,s,t \tag{8}$$

where

- $soc_{n,s,t}$: state-of-charge of every storage
- s : storage technology, $s \in \{$battery, reservoir hydropower plant, e-kerosene tank$\}$
- $\eta_{s,0}$: standing loss per hour to the state-of-charge. For battery, $\eta_{s,0}$ is constant, while for reservoir hydropower plant and e-kerosene tank, no standing losses are assumed ($\eta_{s,0} = 0$).
- $e^{\text{inflow}}_{n,s,t}$: natural inflow to the storage. Only reservoir hydropower plant has time-dependent inflow for each node (cf.[S19]).
- η^{charge}_{s} : charging efficiency. A reservoir hydropower plant operates under the setting of $\eta^{\text{charge}}_{s} = 0$, while a constant value applies to both battery storage and the e-kerosene tank.
- $e^{\text{charge}}_{n,s,t}$: dispatch of power charging at node n
- $\eta^{\text{discharge}}_{s}$: discharging efficiency – set to a constant value
- $e^{\text{discharge}}_{n,s,t}$: dispatch of power discharging at node n.

η^{charge}_{s} and $\eta^{\text{discharge}}_{s}$ determine losses and signify that storage is only charged during periods of excess system power supply and depleted during periods of insufficient power production by generators and import options[S18].

In PyPSA-Brazil, the storage energy capacity, represented by $h^{\max}_{s} \cdot E_{n,s}$, is optimised depending on the storage power capacity $E_{n,s}$. The Energy to Power (E2P) ratio, denoted as $h^{\max}_{s}$, is the fixed duration during which the stored energy can be fully charged or discharged at maximum power[S21].

$$0 \leqslant soc_{n,s,t} \; \leqslant \; h_s^{\max} \cdot E_{n,s} \tag{9}$$

The power capacity of the storage $E_{n,s}$ can be expanded but should be within the upper and lower limits:

$$\underline{E}_{n,s} \leqslant E_{n,s} \; \leqslant \; \overline{E}_{n,s} \tag{10}$$

where

$\underline{E}_{n,s}$: lower bound. For battery and e-kerosene tanks, $\underline{E}_{n,s} = 0$ is set. For hydro reservoirs, however, $\underline{E}_{n,s}$ signifies the installed capacity of all sizes of hydropower plants in the base year, derived from Deng et al.[S19].
$\overline{E}_{n,s}$: upper bound. $\overline{E}_{n,s} = \infty$ is designated for battery and e-kerosene tanks, while for reservoir hydropower, $\overline{E}_{n,s}$ represents the sum of the installed and planned capacity from the Brazilian Ten-Year Energy Plan[S22] (cf. Deng et al.[S19]).

Due to the annual periodicity of demand and seasonal generation patterns, it makes sense to assume cyclic states of charge when optimising a full year[S18]. In this way, storage can be used efficiently at the beginning of the modelled time horizon and avoid the depletion in the end, $soc_{n,s,t=0} = soc_{n,s,t=T} \quad \forall\, n,s.$

B.2.5 E-kerosene generation

The modelling of e-kerosene production employs the `Link` component in PyPSA framework. It is assumed that the capacity expansion of the e-kerosene production unit relies exclusively on a cost basis, setting $0 \leqslant P_{n,k} < \infty \quad \forall\, k.$

The dispatch of e-kerosene generation is not only constrained by its rated capacity but may also be limited by conditions under which it must operate and the availability:

$$\underline{p}_{n,k,t} \cdot P_{n,k} \; \leqslant \; p_{n,k,t} \; \leqslant \; \overline{p}_{n,k,t} \cdot P_{n,k} \quad \forall\, n,k,t \tag{11}$$

where

k : energy conversion technology, $k \in \{\text{e-kerosene production unit}\}$
$P_{n,k}$: capacity of energy conversion
$p_{n,k,t}$: dispatch of energy conversion
$\underline{p}_{n,k,t}$: must-run factor. It is set to 0, $\underline{p}_{n,k,t} = 0$, implying that the conversion from electricity to e-kerosene is a unidirectional process, without any mandatory operational levels.
$\overline{p}_{n,k,t}$: availability factor. It is set as $\overline{p}_{n,k,t} = \eta_k$, where η_k denotes the conversion efficiency of the e-kerosene production unit.

B.2.6 Supply of biokerosene and conventional kerosene

In the model, the supply of biokerosene and conventional kerosene is represented using the `Generator` component in PyPSA framework. It is assumed that there are no capacity limits, hence, setting $0 \leqslant \; G_{n,r} < \; \infty$ (cf. Equation (7)). This implies that the supply amount per hour depends entirely on the marginal cost of providing biokerosene and conventional kerosene, which is measured in €/MWh.

B.2.7 Additional constraints

The constraints in Equations (3) to (11) primarily represent technical restrictions. However, to ensure that the optimisation problem produces feasible solutions, additional constraints can be implemented.

One such constraint involves limiting the total CO_2 emissions ensuring that they do not exceed a specified budget, denoted as Γ_{CO_2}:

$$\sum_{n,r,t} \frac{1}{\eta_{n,r}} \; g_{n,r,t} \; \cdot \; \rho_r \; \leqslant \; \Gamma_{CO_2} \quad \leftrightarrow \quad \mu_{CO_2} \tag{12}$$

where

r : technology, $r \in \{\text{fossil generators, conventional kerosene supply}\}$.
$\eta_{n,r}$: generator efficiency at node n for technology r
$g_{n,r,t}$: generator dispatch at time t
ρ_r : fuel-specific emissions, measured in units of $CO_2\,t/MWh$. This emission rate is assumed to apply only to the supply of conventional kerosene.
Γ_{CO_2} : predetermined budget of CO_2 emissions
μ_{CO_2} : KKT multiplier, also referred to as the shadow price. It indicates the marginal cost of emitting an additional tonne of CO_2. It can also be interpreted as the additional cost required to achieve the CO_2 emissions reduction target.

In our scenario analysis, there is an option to limit the contribution of e-kerosene to a specific fraction of the total kerosene demand. This results in a constraint where the combined supply of biokerosene and conventional kerosene must exceed a certain proportion (γ) of the total kerosene demand, $d_{n,t}^{\text{kerosene}}$:

$$\sum_{n,t} g_{n,r,t} = \gamma \cdot \sum_{n,t} d_{n,t}^{\text{kerosene}} \tag{13}$$

where $r \in \{\text{conventional kerosene supply, biokerosene supply}\}$. For instance, in the "100% e-kerosene supply" scenario, $\gamma = 0$ is set.

B.3 Assumptions about e-kerosene production route

The e-kerosene production chain has a wide choice of technologies that correspond to each processing step, namely, the provision of H_2 and CO_2, the synthesis and upgrading[S23]. This section outline herein the assumptions and simplifications employed in the modelling of e-kerosene production within the PyPSA-Brazil model, which we refer to as the "e-kerosene production link" in Main Text Figure 5.

We utilise a simplified representation of the e-kerosene production plant, as opposed to the detailed component modelling delineated in Sherwin[S24]. The model relies on conversion efficiencies and techno-economic parameters derived from Schmidt et al.[S25] to emulate the technical behaviour. The plant features a conversion efficiency from electricity to e-kerosene of 0.42 and includes a plant configurations of low-temperature electrolysis – Alkaline Electrolysis (AEL) or Proton Exchange Membrane Electrolysis (PEM) – hydrogen storage, CO_2 sourced from Direct Air Capture (DAC), and a Fischer–Tropsch (FT) pathway comprising FT synthesis, Reverse Water-gas Shift (rWGS), and hydrocracking/isomerisation.

In the provision of CO_2, fossil CO_2 using carbon capture and utilisation is elected not to used, as it falls short as a long-term, carbon-neutral solution[S26]. Additionally, while CO_2 could be obtained from concentrated sustainable sources such as biomass combustion, organic residues, and bio-ethanol production from sustainably produced sugars or starches[S27], these options call for access to extensive biomass data. As such, we assume that CO_2 is sourced exclusively from the atmosphere via DAC and is universally available in Brazil.

The assumption is made that high-purity water needed for FT-based e-kerosene production is readily available in Brazil and may not pose a technological challenge in Brazil. This is due to the relatively lower water demand compared to biokerosene production by Hydro-processed Esters and Fatty Acids (HEFA) process or Alcohol-to-jet (ATJ)[S23]. The ready availability of both groundwater[S28] and surface water[S29], coupled with Brazil's status as one of the world's leading nations in terms of seawater availability[S30] and the successful water scarcity management[S31], implies that the pre-treatment process of water or the cost thereof is deemed negligible.

The focus of our research is the FT synthesis pathway, a prevalent process in large-scale industrial applications for producing liquids from natural gas or coal[S32]. Although methanol synthesis is capable of yielding e-kerosene, so far, the first-of-its-kind aviation fuel specification – namely, the American Society for Testing and Materials (ASTM) D7566 standard – exclusively specifies this synthetic kerosene derived from the FT process[S33]. A noteworthy assumption in our study is the potential for altering future blending ratios of e-kerosene with crude oil-based kerosene[S32, S34], speculating that they may no longer be limited to the 50%, currently prescribed by ASTM regulations.

We explicitly model the energy system with generation, storage, transmission and demand for electricity supply for e-kerosene production using PyPSA-Brazil model. By considering the dynamics of the system, the cost of electricity supply is calculated endogenously at the federal-state level in Brazil.

The PyPSA-Brazil model posits that the e-kerosene production results in an Emissions Reduction Factor (ERF) of 100%. ERF measure the net life-cycle CO_2 benefits of Sustainable Aviation Fuel (SAF) by accounting for the CO_2 savings from feedstock production or growth, and incorporating the emissions incurred during fuel production[S35]. 100% ERF indicates that there is no net carbon loss between the stages of emissions and capture, which is also referenced by Micheli et al.[S36]. However, Micheli et al.[S36] adopt a more conservative stance, assuming 5% loss within the closed carbon cycle. Such a loss translates to emissions ranging from 0.9-4.0 $g\,CO_2e/MJ$, on the condition that electricity is sourced from wind or solar energy. In addition,

the Air Transport Action Group explores sustainable trajectories for aviation by assuming ERF of 70-100% for SAF[S35]. This is indicative of the industry's direction towards mitigating carbon emissions.

It is imperative to underscore that focusing solely on achieving a closed carbon cycle with regard to CO_2 emissions does not address the broader environmental impacts. Specifically, aircraft emissions in the upper atmosphere, including water vapour, aerosols, and nitrogen oxides (NOx), have a significantly more detrimental effect on the climate compared to CO_2 emissions at lower altitudes[S37,S38]. These non-CO_2 impacts, often overlooked, merit greater consideration.

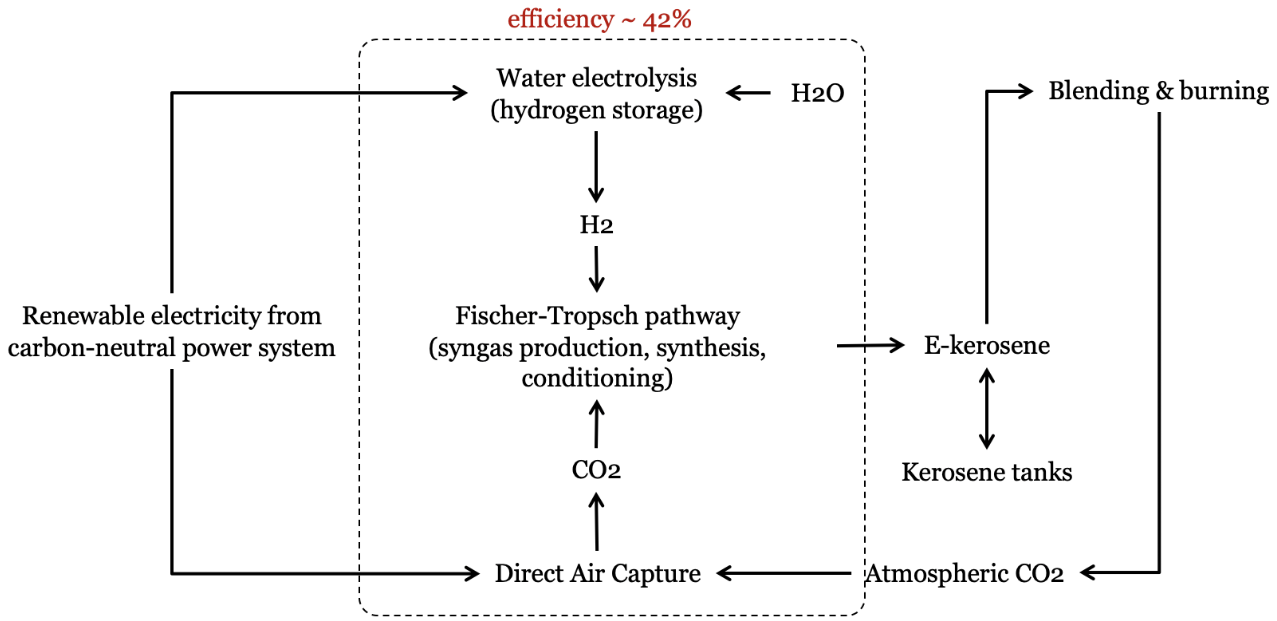

Figure S1. E-kerosene production unit considered (own illustration inspired by Schmidt and Weindorf[S23], König et al.[S39], and Drünert et al.[S32]).

C Data

C.1 Power sector

The inputs and the assumptions underlying the model of the Brazilian power system are well documented in Deng et al.[S19], where the open, spatially resolved, harmonised data set of the Brazilian energy system is introduced. PyPSA-Brazil adopts 2019 as the base year in this study, ensuring that all input data are from that year.

Stored capacity for reservoir hydropower plant We assume that the hydropower plants are of reservoir type, with their energy capacity determined on the E2P ratio and power capacity (cf. Equation (9) and Deng et al.[S19]). We derive the E2P ratio from the data set of stored energy capacity for each electric region in the National Interconnected Network (Portuguese: Sistema Interligado Nacional, SIN), released by National Electricity System Operator (Portuguese: Operador Nacional do Sistema Elétrico, ONS)[S40]. The stored capacity of hydropower is about 357,548,409 MWh. This figure, accumulated daily across the four electric regions, is presented in MWmês. For clarity, we apply the conversion: 1 MWmês = 720 MWh/month. This regional storage energy capacity then undergoes conversion to the federal-state level:

$$h_s^{\max} = \frac{E_{n,s}}{\sum_n E_{n,s}} \cdot \frac{\mathscr{S}_{R,s}}{\sum_n E_{n,s}} \tag{14}$$

where

h_s^{max} : E2P ratio assumed, in the unit of h
s : storage technology, $s \subseteq$ reservoir hydropower plant
n : node of the model – Brazilian federal state, $n \in \mathcal{N}$, $\mathcal{N} = 1, 2, \ldots, 27$
$E_{n,s}$: power capacity at federal state n, in unit of MW
R : electric regions defined by SIN (cf. Deng et al.[S19])
$\mathscr{S}_{R,s}$: stored energy capacity at the region R, in unit of MWh.

C.2 Cost assumptions

The financial and technical assumptions used in this study, such as investment costs, Variable Operation and Maintenance (VOM) costs, Fixed Operation and Maintenance (FOM) costs, efficiency, and lifetime, are based on insights from the scientific literature and are presented in the Table S5. The capital investment of each technology is annualised with a discount rate of 8% over the economic lifetime used in Equation (2).

Capital investment in transmission lines comes from historical auctions with units of R$/MW/km[S41]. The capital investment costs are derived by interpolating representative line lengths for Alternating Current (AC) and High Voltage Direct Current (HVDC) lines, considering the cost of converters and transformers for each historical auction.

Table S5. Technology cost and lifetime assumptions for 2050 (VOM, (FOM)).

Technology	Parameter	Value	Unit	Source
Unit conversion	discount rate[1]	0.08	per unit	S42
	jet fuel density[2]	775	kg/m^3	S33
	jet fuel heat	42.8	MJ/kg	S33
	euro to dollar[3]	1.119	$/€	S48
	euro to real[3]	4.413	R$/€	S49
	ton to kg	1,000	kg/t	S50
	m^3 to L	1,000	L/m^3	S50
	MWh to MJ	3,600	MJ/MWh	S50
Solar PV[4]	FOM[5]	1.6	%/year	S51
	VOM[6]	0.01	€/MWh	S16
	investment	490,000	€/MW	S51
	lifetime	40	years	S51
Offshore wind[7]	FOM[8]	1.8	%/year	S51
	VOM	2.4	€/MWh	S51
	investment[9]	1,780,000	€/MW	S51
	lifetime	30	years	S51
Onshore wind[10]	FOM[11]	1.2	%/year	S51
	VOM	1.22	€/MWh	S51
	investment[12]	960,000	€/MW	S51
	lifetime	30	years	S51

Continued on next page

Technology	Parameter	Value	Unit	Source
Hydro reservoir	FOM	2	%/year	S43
	efficiency	0.9	per unit	S43
	investment[13]	1,565,000	€/MW	S43
	lifetime	80	years	S43
	max hour[14]	18	h	S52
Biomass thermal	FOM[15]	1.7	%/year	S42
	VOM	2.1	€/MWh	S51
	investment[15]	1,200,000	€/MW	S42
	lifetime	25	years	S51
	efficiency[16]	0.39	per unit	S53
	fuel	7	€/MWh	S54
AC line	investment[17]	6286.5	R$/MW/km	S41
	FOM	2	%/year	S55
	lifetime	40	years	S55
HVDC line	investment[17]	864.56	R$/MW/km	S41
	FOM	2	%/year	S55
	lifetime	40	years	S55
Battery storage[18]	investment[19]	75,000	€/MWh	S56
	max hour[20]	6	h	S53
	loss[21]	0.004	per unit	S56
	lifetime[22]	30	years	S56
Battery inverter[23]	charge efficiency[24]	0.985	per unit	S56
	discharge efficiency	0.975	per unit	S56
	lifetime[22]	30	years	S56
	FOM[25]	0.9	%/year	S56
	VOM	1.6	€/MWh	S56
E-kerosene unit[26]	investment[27]	2,000,000	€/MW	S44
	VOM[28]	7.4	€/MWh	S51
	lifetime[29]	25	years	S57
	efficiency[30]	0.42	per unit	S44
Kerosene tank[31]	efficiency	0.9	per unit	S58
	investment	0.098	€/L	32
	lifetime	30	years	S58
	max hour	144	h	S59

Continued on next page

Technology	Parameter	Value	Unit	Source
Jet fuel	CO_2 emissions[33]	3.16	$kg\,CO_2/kg\,Jet$	S47

[1] The value is sourced from [S42, Anoexos, p. A-3].
[2] The value represents the lower limit of aviation fuel density, ranging from 775-840 kg/m^3.
[3] The 2019 annual average is used.
[4] The value comes from "sheet 22 Photovoltaics medium".
[5] The value results from dividing "Fixed O&M" by "Specific investment, total system" in the raw data.
[6] A value of 0.01 adjusts the curtailment order of renewable technology[S16].
[7] The value originates from "sheet 21 Large wind turbines offshore".
[8] This is a ratio of "Fixed O&M" to "Variable O&M" derived from the original data.
[9] The value is obtained by deducting "Nominal investment grid connection costs" from "Investment costs".
[10] The value is sourced from "sheet 20 Large wind turbines on land".
[11] This is a ratio of "Fixed O&M" to "Nominal investment" as observed in the raw data.
[12] This refers to "Nominal investment".
[13] This refers to "Reservoir repowered" from [S43, p. 27, 2.5 Hydro].
[14] The PyPSA-Brazil model updates this threshold for each state based on stored capacity.
[15] The value originates from [S42, p. A-6].
[16] This value is extracted from the "Biomass CHP" in the supplementary material.
[17] This value is derived from the source data by dividing the investment cost (in R$) by transfer capacity (in MW) and line length (in km). For multiple capacities, the smallest is chosen, ignoring voltage differences.
[18] The value comes from "sheet 180 Lithium-ion battery".
[19] This refers to the "Energy storage expansion cost (0.075 M€2015/MWh)" from the raw data.
[20] This refers to the E2P ratio.
[21] The value of "Energy losses during storage(%/day)" from the raw data is divided by 24 for consideration.
[22] This refers to "Technical lifetime".
[23] The value is sourced from "sheet 180 Lithium-ion battery".
[24] This refers to the "Round trip efficiency DC (discharge) charging efficiency (%)".
[25] The ratio is derived from "Fixed O&M" to "Output capacity expansion cost investment·100" in the raw data.
[26] This considers a range of technologies and processes, including low-temperature electrolysis (AEL or PEM), H_2 storage, CO_2 from DAC, FT pathway (FT synthesis, rWGS and hydrocracking, isomerization).
[27] The value is a division of "Total[M€]" and "Fuel output" in [S44, Table 2.].
[28] The value combines "Fix O&M" and "Variable O&M" from "sheet 102 Power to Jet Fuel".
[29] This refers to the "Technical lifetime" from "sheet 102 Power to Jet Fuel".
[30] The value is sourced from "Efficiency[%]" from [S44, Table 2.].
[31] This is assumed to be of an aboveground petroleum storage tank type.
[32] The original value of £7,266[S45] is converted from GBP to EUR based on the 2019 annual average[S46], and then divided by 100,000L to be the desired value.
[33] The value is sourced from [S47, Table 1]. The GHG emissions is 3.166 $kg\,CO_2e/kg\,Jet$.

C.3 Nodes in PyPSA-Brazil

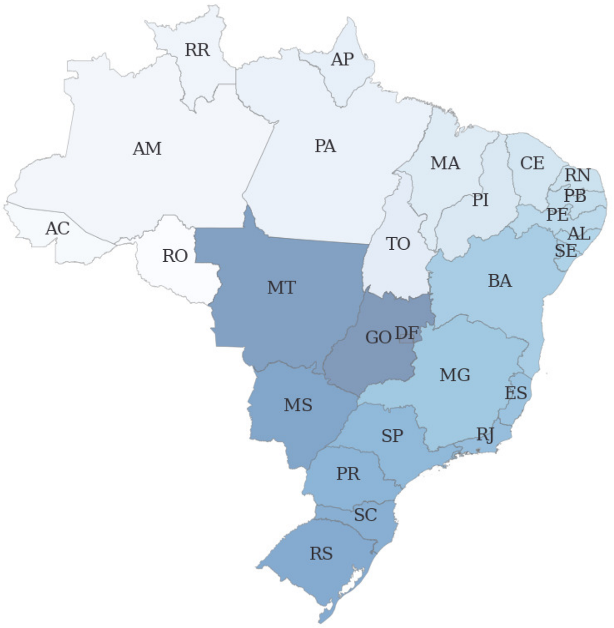

Code	Name	Code	Name
AC	Acre	PB	Paraíba
AL	Alagoas	PR	Paraná
AP	Amapá	PE	Pernambuco
AM	Amazonas	PI	Piauí
BA	Bahia	RJ	Rio de Janeiro
CE	Ceará	RN	Rio Grande do Norte
DF	Distrito Federal	RS	Rio Grande do Sul
ES	Espírito Santo	RO	Rondônia
GO	Goiás	RR	Roraima
MA	Maranhão	SC	Santa Catarina
MT	Mato Grosso	SP	São Paulo
MS	Mato Grosso do Sul	SE	Sergipe
MG	Minas Gerais	TO	Tocantins
PA	Pará		

Figure S2. 27 regions defined – Brazilian federal states – used in PyPSA-Brazil[S19]

C.4 Aviation sector

C.4.1 Kerosene demand

In the model, aviation kerosene demand for 2050 is an hourly time series of historical federal-state kerosene demand statistics scaled up to projected national fuel demand in 2050.

The model incorporates kerosene demand from both domestic and international flows at civil airports in 2019. Brazil's National Civil Aviation Agency (Portuguese: Agência Nacional de Aviação Civil, ANAC) publishes extensive air transport statistics with an array of 110 variables[S60]. These variables capture critical parameters such as the number of passengers, cargo and postal traffic, distance flown, and fuel consumption, among others, for each stage of flight and each airline. They reflect key industry indicators, like RTK, Revenue Passenger Kilometers, Available Seat Kilometers, and Available Tonne Kilometers.

The model assumes flights departing from Brazilian airports are refuelled within Brazil, thereby constituting the kerosene demand for each airport. As a result, four attributes of the original air transport data set (as detailed in Table S6) are used. By accounting for the location of the refuelling airport, the kerosene demand is aggregated by each federal state. The model presumes that the departure time of the aircraft corresponds to the refuelling date, which yields a daily kerosene demand pattern. This daily time series is then averaged out to derive an hourly demand profile (presented in Figure S3).

Table S6. Attributes used in PyPSA-Brazil from the air transport statistics[S60].

PyPSA-Brazil	Original data set	Unit	Type	Explanation
`origin_state_name`	`sg_uf_origem`	—	string	abbreviation of Brazilian federal state, in total 27 federal units
`departure_date`	`dt_partida_real`	—	string	time stamp of the real take-off date of the state, `yy:MM:dd hh:mm:ss`
`fuel_consumption`	`lt_combustivel`	L	float	the kerosene demand

In 2019, the kerosene demand is 4,286.6 ML (approximately 39.5 TWh), with the highest demand found in the federal state of SP(39.9%), followed by RJ and DF (7.9% each), PE (5.2%), BA (4.8%), CE (4.5%), MG (4.4%), RS and PR (3.2% each). Historical kerosene demand is available for 2000-2020, but the model only employs the time series for the base year 2019 (cf. Figure S4).

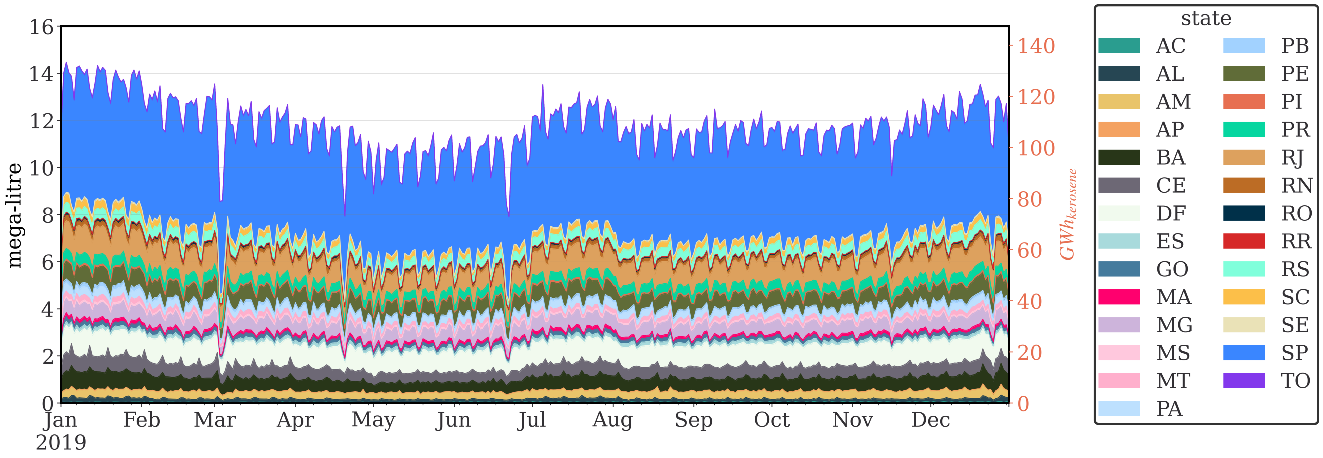

Figure S3. 2019 kerosene demand by federal states. The left y-axis is in units of the original data, and the right axis is in units converted to GWh according to Table S5.

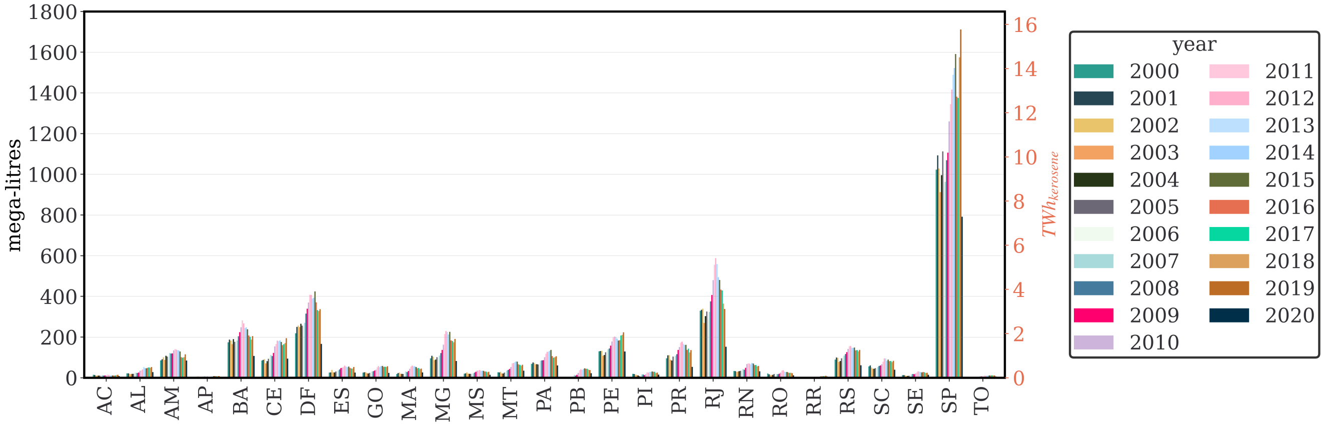

Figure S4. Two-decade kerosene demand by federal states (2000-2020): dual y-axis display of original and converted units (TWh) as per Table S5.

The ANAC releases official annual demand forecasts for domestic and international flights up to 2050, distinguished by two scenarios – "without mitigation" and "with mitigation"[S61]. The projection for the period 2019-2050 on total annual national kerosene demand is determined using monthly Revenue Tonne Kilometer (RTK) and kerosene consumption statistics from 2010-2018, as well as the anticipated progress in fuel efficiency[S62]. In the "with mitigation" scenario, Brazil's total kerosene consumption is expected to reach 13.2 million tons in 2050[S61], representing an overall growth of 217.5% and an average annual growth of 3.7% from 2018 to 2050. This projection of kerosene consumption of 13.2 million tons (around 157 TWh) for 2050 is integrated into the PyPSA-Brazil model, which is approximately four times higher than the consumption level in the base year of 2019.

C.4.2 Kerosene supply

According to Figure 5 of the Main Text, the aviation sector consumes kerosene that is synthetically produced with e-kerosene, fossil origin, or biokerosene.

Supply of conventional kerosene The supply of conventional kerosene depends purely on the cost of supply.

The National Agency for Petroleum, Natural Gas and Biofuels (Portuguese: Agência Nacional do Petróleo, Gás Natural e Biocombustíveis, ANP) regularly updates the fuel distribution prices at a national, regional, and federal state level[S63]. The state-level data are incorporated into PyPSA-Brazil, using regional data when values are absent for certain states such as SE and AP. It is assumed that the conventional kerosene price (R$/L) in 2019 is the marginal production cost in each state in 2050. An average hourly price is then determined from the monthly prices (cf. Figure S5) since PyPSA-Brazil considers a

time-dependent marginal cost. With the carbon emissions from conventional kerosene generation, a carbon price is introduced for Brazil to achieve carbon neutrality (cf. Section C.6).

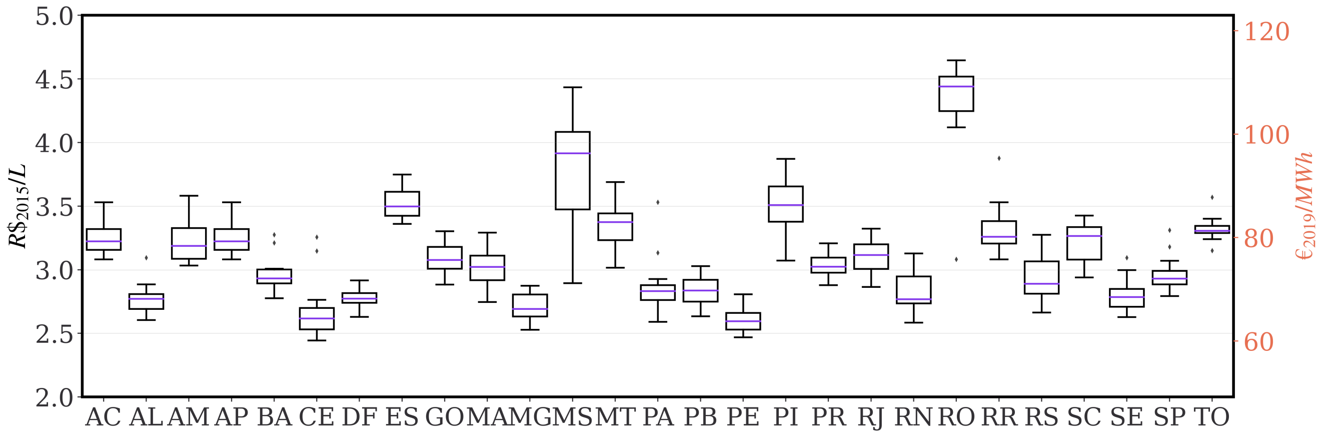

Figure S5. Box plot of monthly kerosene distribution price across federal states in 2019: input data of PyPSA-Brazil model processed from original source[S63], with the raw and converted units of €/MWh (cf. Table S5).

Supply of biokerosene PyPSA-Brazil model posits that the biokerosene potential for 2050 is measured by its production cost, as appraised by Cervi et al.[S64]. This assessment employs techno-economic assessment and spatial data analysis to examine the potential of 13 distinct production routes in 2030 at a spatial resolution of 5 km (cf. Figure S6). These routes comprise eight first-generation biomass – maize, sugarcane, sorghum, eucalyptus, soybean, sunflower, palm, and macaw – and five conversion pathways, including ATJ, HEFA, FT, Direct Fermentation of Sugars to Hydrocarbons, and Hydrotreated Depolymerized Cellulosic Jet. Cervi et al.[S64] assume that the land used for biokerosene production is leftover – it is not utilised for other purposes, such as forest plantations and urbanisation, and it is not set aside for conservation areas.

Cervi et al.[S64] provide data in $/t, which is converted to €/MWh for integration into PyPSA-Brazil. The resolution provided by Cervi et al.[S64] for the cost of biokerosene production is 5 km, whereas PyPSA-Brazil utilises a coarser spatial resolution corresponding to federal states. In PyPSA-Brazil, the production cost of biokerosene for each federal state is determined by calculating 10%, 25%, 50% percentile of the cost within 5 km × 5 km cell, as depicted in Figure S7. For scenario analysis, the input to the model indicates that the production cost at the federal-state level ranges from 69.5-149.2 €/MWh at a low level, 104.2-234.6 €/MWh at a medium level, and 147.8-725.6 €/MWh at a high level.

As the biomass used in Cervi et al.[S64] (maize, sugarcane, sorghum, eucalyptus, soybean, sunflower, palm and macaw) is of the biogenic type, the life-cycle GHG emissions of biokerosene are set to be carbon neutral[S65], signifying its environmental sustainable character.

C.5 Carbon emission cap

In PyPSA-Brazil, total carbon emissions across sectors are limited so as not to exceed a predetermined budget, represented by Γ_{CO_2} as shown in Equation (12). In compliance with Brazil's Intended Nationally Determined Contributions, the nation is committed to cutting GHG emissions to 50% below the levels recorded in 2005 by the year 2030, and further attaining climate neutrality by 2050 [S66, p. 1]. This commitment translates into an average annual reduction rate of 2%. The scarcity of sector-specific statistics and emission budgets, however, necessitates certain assumptions for modelling purposes.

As a reference, in 2006, the power sector was responsible for the emissions of 26.42 Mt CO_2e[S67]. Since data for 2005 is not available, we assume a consistent 2% reduction rate from 2006 to 2050. This assumption yields an 88% reduction by 2050 based on 2006 emissions, setting a budget of 3.17 Mt CO_2e. This budget represents GHG emissions, and in the context of PyPSA-Brazil, it is regarded as the carbon emission budget for the power sector.

Regarding the aviation sector, the ANAC has estimated the carbon emissions for the period 2019-2050, drawing upon the projected kerosene consumption for domestic and international flights. This calculation, underpinned by the assumption of kerosene demand being exclusively met by conventional kerosene, applies a carbon emission factor of 3.16 kg CO_2/kg Jet [S61, p. 32]. Consistent with the global mitigation measure to secure carbon-neutral growth in aviation from 2020, and net-zero carbon emissions by 2050 [S68, p. 1-2], PyPSA-Brazil model postulates that Brazil adopts an emissions budget for 2050 equivalent to the projection for 2020[S61], amounting to 14.2 million tonnes. This budget of 14,221,089.4 tonnes pertains to carbon emissions. Consequently, the model permits up to 34% of the demand to be satisfied by conventional kerosene, assuming a total kerosene consumption of 13.2 million tonnes in Brazil by 2050.

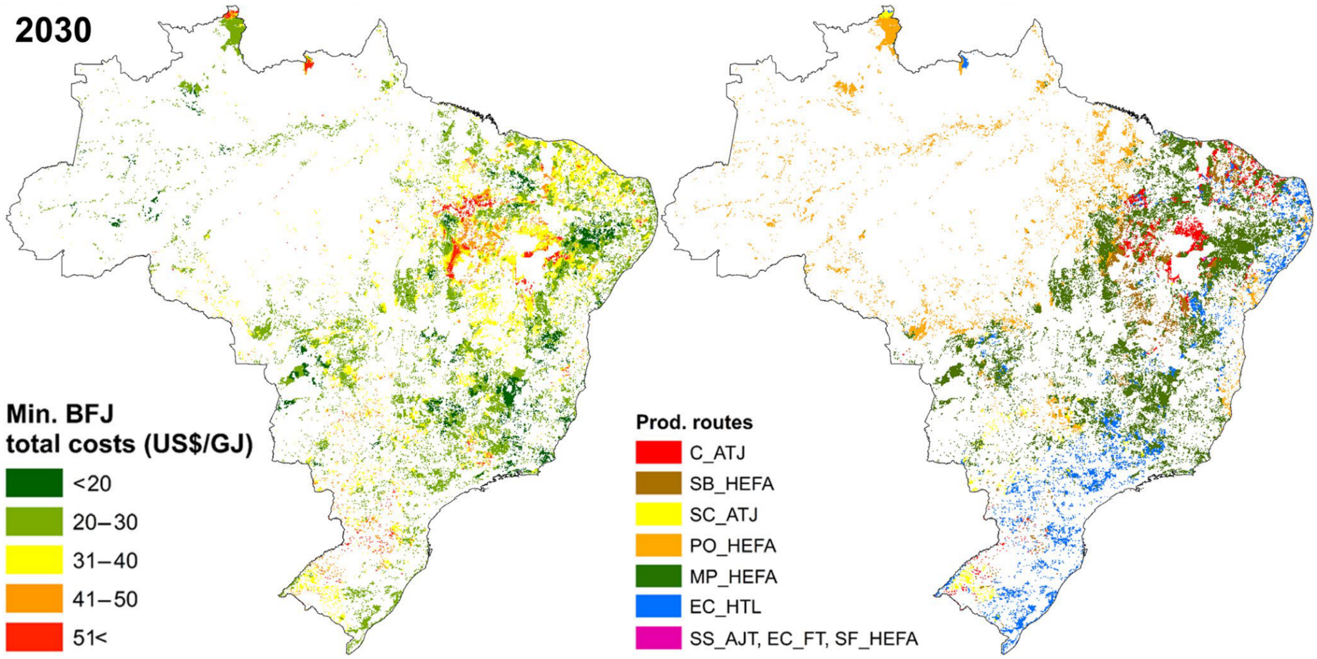

Figure S6. Minimum biokerosene costs [S64, Figure 9]. Corn ethanol via alcohol-to-jet (C_ATJ), soybean oil via hydroprocessed esters and fatty acids (SB_HEFA), sugarcane ethanol via ATJ (SC_ATJ), palm oil via HEFA (PO_HEFA), macaw oil via HEFA (MP_HEFA), eucalyptus via hydrothermal liquefaction (EC_HTL), sweet sorghum ethanol via ATJ (SS_ATJ), eucalyptus via Fischer–Tropsch (EC_FT), sunflower oil via hydroprocessed esters and fatty acids (SF_HEFA).

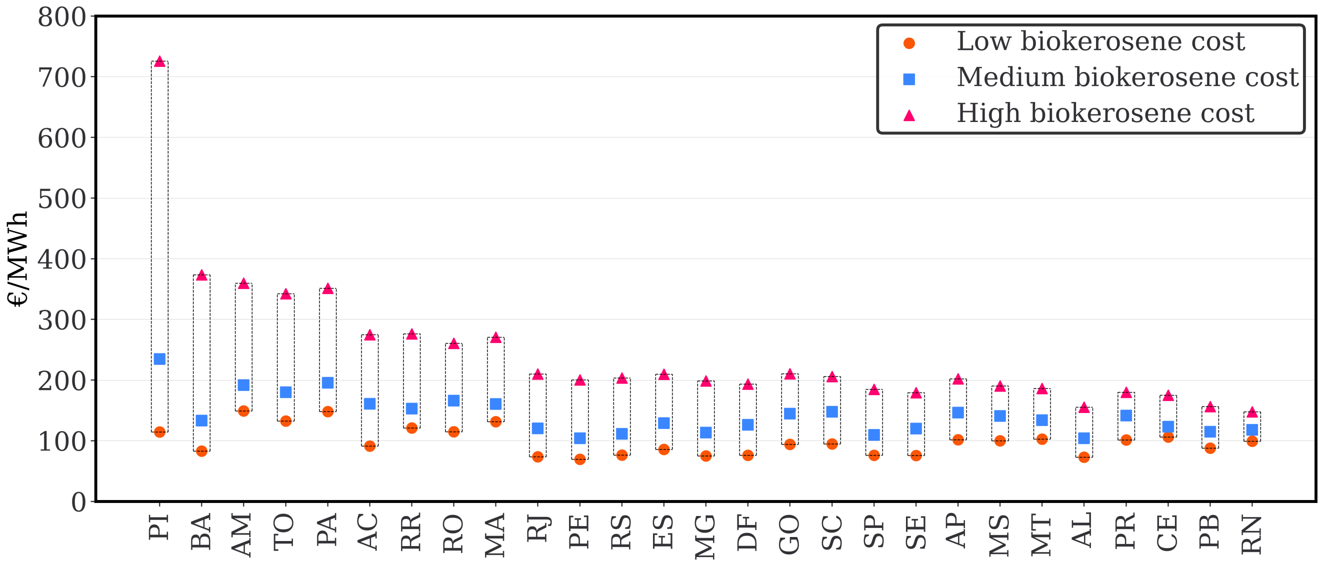

Figure S7. Input assumptions of biokerosene production costs for the federal states used in PyPSA-Brazil. Each marker represents the input assumption – the percentile of biokerosene production costs for pixel points within the federal state. The lower the percentile, the lower the presumed production cost. The range of variation orders the values.

C.6 Carbon pricing range

Carbon pricing is an instrumental policy tool that places a charge on GHG emissions, thereby encouraging governments and industries to modify their production, consumption, and investment habits in favour of low-carbon growth[S69]. As of 2022, Brazil has been evaluating the introduction of carbon pricing, though it has not yet been established [S69, p. 12, 55]. Notably, the current carbon pricing in effect regulates only the CO_2 emissions from aviation[S70]. For instance, the EU's Emission Trading System (EU ETS) focuses on CO_2 emissions because the scientific understanding of non-CO2 effects (such as NOx, water vapour, soot, sulphates, and contrails) is not yet sufficiently mature to formulate comprehensive policies[S70,S71]. For this reason, PyPSA-Brazil limits the application of carbon budgets and carbon pricing to CO_2 emissions.

In our scenario analysis, carbon pricing is primarily applied to the combustion of jet fuel, which leads to CO_2 emissions. The analysis begins with the implementation of a carbon emissions budget (cf. Section C.5), and in PyPSA-Brazil, this corresponds to an initial carbon price of 160 €/t.

The analysis then explores a range of carbon prices for the year 2050, extending from 160 €/t to 1000 €/t. This range includes specific price points such as 200 €/t, 260 €/t, 320 €/t, and 500 €/t. This range is chosen to account for potential extreme shifts in carbon pricing, possibly due to drastic policy changes or unforeseen global events impacting the carbon market in 2050[S72]. The assumed range of carbon prices is in line with the IPCC's mitigation pathway of limiting the median warming to below 1.5 °C by 2100. The selected carbon prices fall within the range specified by two IPCC scenarios: the "low overshoot pathway", which holds a 50-67% likelihood of a temporary early overshoot of 1.5 °C, and the high overshoot pathway, having an over 67% probability of an early temporary overshoot[S72].

D Equations for analysis

D.1 Average system cost

In our study, the Average System Cost (ASC) is employed as a metric that quantifies the average optimised total system cost per unit of energy generated within an energy system [S18, Fig. 2]. Employing ASC as a metric enables the insightful comparison of the relative costs associated with energy supplies across various system designs through a given equation:

$$\text{ASC} = \frac{\mathscr{C}^*}{\Gamma^*} \tag{15}$$

where

$\mathscr{C}^*$: optimised total system cost, €, computed as outlined in Equation (1)
Γ^* : optimised total dispatch, MWh. It includes the amount of energy produced by renewable generation technologies and the supply of kerosene in the system.

D.2 Levelised cost of energy

The Levelised Cost of Electricity (LCOE) assesses the costs associated with electricity generation from a single technology, with the costs being the net present sum of investment, fuel, operational and maintenance costs[S17,S73]. We use LCOE to compare the generation cost for each technology among studies through the equation:

$$\text{LCOE}_r = \frac{\sum_n c_{n,r} \cdot G^*_{n,r} + \sum_{n,t} o_{n,r} \cdot g^*_{n,r,t}}{\sum_{n,t} g^*_{n,r,t}} \tag{16}$$

where

LCOE_r : levelised cost of electricity for technology r
r : generation technology
n : node
$c_{n,r}$: annualised capital costs for technology r
$G^*_{n,r}$: optimised installed capacity of generators
$o_{n,r}$: variable costs
$g^*_{n,r,t}$: optimised generation dispatch, MWh.

D.3 Levelised cost of fuel

The Levelised Cost of Fuel (LCOF) refers to the supply of e-kerosene, which is essential for determining its economic viability:

$$\mathrm{LCOF} = \frac{\sum_{n}\{\sum_{k,t}(c_{n,k}\cdot P_{n,k} + o_{n,k}\cdot p_{n,k,t}) + \sum_{s,t}(c_{n,s}\cdot E_{n,s} + o_{n,s}\cdot e_{n,s,t}) + \overline{\lambda_n^{\text{electricity}}}\cdot \Gamma_n^{\text{electricity}}\}}{\sum_{n}\Gamma_n^{\text{e-kerosene}}} \tag{17}$$

where

n : node, $n \in \mathscr{N}$, $\mathscr{N} = 1,2,\ldots,27$
k : energy conversion technologies, $k \in \{\text{e-kerosene production unit}\}$
t : hour
$c_{n,*}$: annualised capital expenditures
$P_{n,k}$: installed capacity of technology k at node n
$o_{n,*}$: variable operational expenditure
$p_{n,k,t}$: e-kerosene dispatch at hour t at node n
s : storage technology, $s \in \{\text{e-kerosene tank}\}$
$E_{n,s}$: power capacity of storage technology s at node n
$e_{n,s,t}$: storage dispatch of e-kerosene tank
$\overline{\lambda_n^{\text{electricity}}}$: median KKT multiplier of electricity at node n (cf. Equation (3))
$\Gamma_n^{\text{electricity}}$: total electricity consumption for e-kerosene production at node n
$\Gamma_n^{\text{e-kerosene}}$: total e-kerosene supply at node n.

D.4 Export cost

The cost of export is defined as the relative deviation compared to the reference scenario without export:

$$\mathscr{E}_{\beta,\delta} = \frac{\mathscr{C}_{\beta,\delta} - \mathscr{C}^{\text{Ref.}}}{d_{\beta,\delta}^{\text{kerosene}} - d^{\text{Ref.,kerosene}}} = \frac{\mathscr{C}_{\beta,\delta} - \mathscr{C}^{\text{Ref.}}}{\delta\cdot d^{\text{Ref.,kerosene}}} \tag{18}$$

where

$\mathscr{E}_{\beta,\delta}$: export cost for the scenario given β,δ
β : level of production cost for biokerosene, $\beta \in \{\text{low, medium, high}\}$
δ : additional kerosene demand for export, $\delta \in \{50\%, 100\%, 150\%, 200\%, 400\%, 500\%\}$
$\mathscr{C}_{\beta,\delta}$: total system cost of the scenario given β,δ
$\mathscr{C}^{\text{Ref.}}$: total system cost of the reference scenario
$d_{\beta,\delta}^{\text{kerosene}}$: total kerosene demand of export scenario given β,δ, $d_{\beta,\delta}^{\text{kerosene}} = (1+\delta)\cdot d^{\text{Ref.,kerosene}}$
$d^{\text{Ref.,kerosene}}$: total kerosene demand in the reference scenario, i.e., 157 TWh.

D.5 Export amount of the e-kerosene

The amount of the e-kerosene for export is the absolute difference in the amount of e-kerosene supply between the export scenario and the reference scenario.

$$\mathscr{P}_{\beta,\delta} = p_{\beta,\delta} - p^{\text{Ref.}} \tag{19}$$

where

$\mathscr{P}_{\beta,\delta}$: e-kerosene for export given β,δ
$p_{\beta,\delta}$: generation of e-kerosene given β,δ
$p^{\text{Ref.}}$: generation of e-kerosene in the reference scenario.

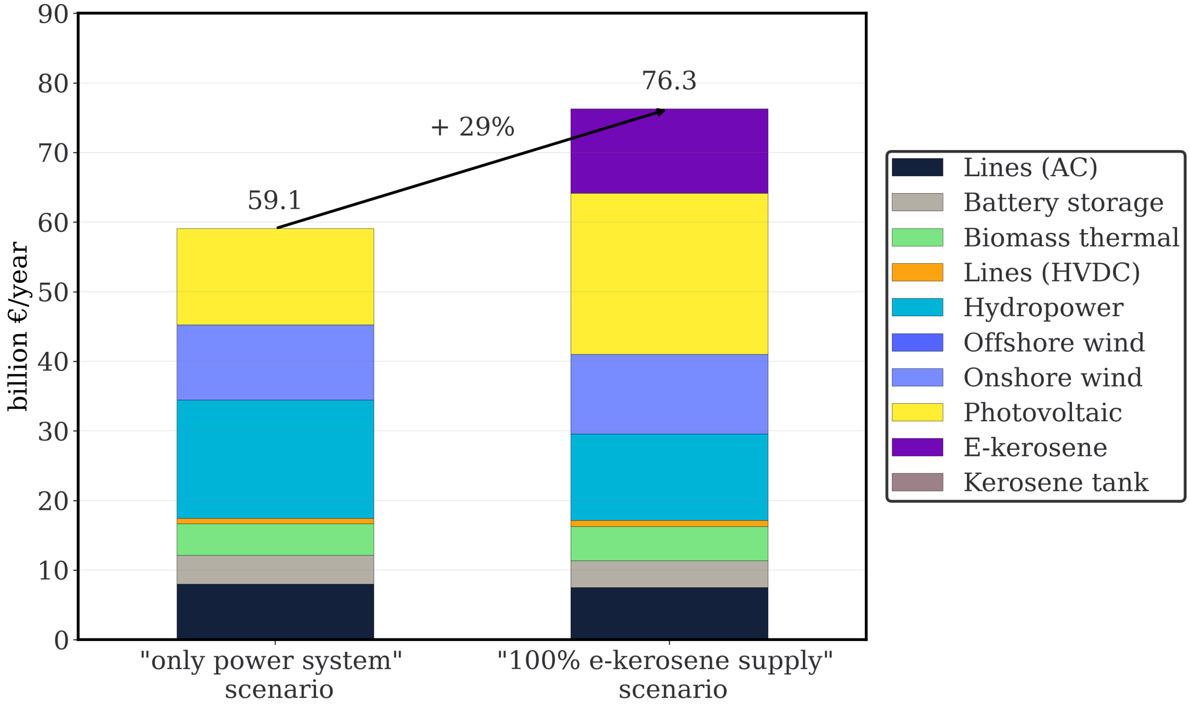

Figure S8. Breakdown of annual total system costs in Brazil's carbon-neutral power system scenarios without ("only power system") and with e-kerosene production integration ("100% e-kerosene supply").

E Further results & discussions

E.1 Results

E.1.1 Annual total system costs

The PyPSA-Brazil model outcomes, displayed in Figure S8, indicate a rise in total system costs, which is driven by the additional provision of e-kerosene (increasing from 59.1 to 76.3 billion €/ year, by 29%). The main contributor to this increase is the further investment in PV installations, which provides 317 TWh. In contrast, hydropower remains relatively stable, while wind onshore (additionally 35 TWh) and biomass (additionally 38 TWh) energy see an uptick. E-kerosene production offers some load-balancing flexibility, leading to a slightly reduced investment in battery storage.

E.1.2 Annual electricity generation

Figure S9 illustrates the additional electricity generation required for producing e-kerosene in a carbon-neutral power system. Specifically, the results accentuate the substantial contribution from PV generation. This is complemented by 35 TWh from onshore wind and 39 TWh from biomass thermal energy. While hydropower experiences a slight rise, contributing to 2 TWh, it pales in comparison to the aforementioned technologies. Offshore generation also contributes but has a minimal impact in both scenarios.

E.2 Discussions

E.2.1 Feasible e-kerosene production in Brazil from abundant renewable potential

As shown in Table S7, São Paulo emerges as the dominant federal state in terms of electricity and kerosene demand, given its stature as the country's largest economy and the most populous state. In contrast, Rio de Janeiro and Distrito Federal have relatively high population densities. São Paulo's low population density might contribute positively in lessening social acceptance issues linked to land use for PV expansion. Distrito Federal, characterised by the highest population density, predominantly relies on importing electricity from neighbouring states. Adjacent to Distrito Federal, Goiás displays moderate electricity demand and relatively low kerosene demand. The lower population density in Goiás positions it favourably for PV expansion and for acting as an electricity conduit or even a powerhouse to other federal states. Minas Gerais follows São Paulo in electricity and kerosene demand, and its low population density might result in fewer social acceptance issues regarding new PV installations. Lower population density can be a factor in diminishing local acceptance conflicts, albeit not a definitive solution[S74,S75].

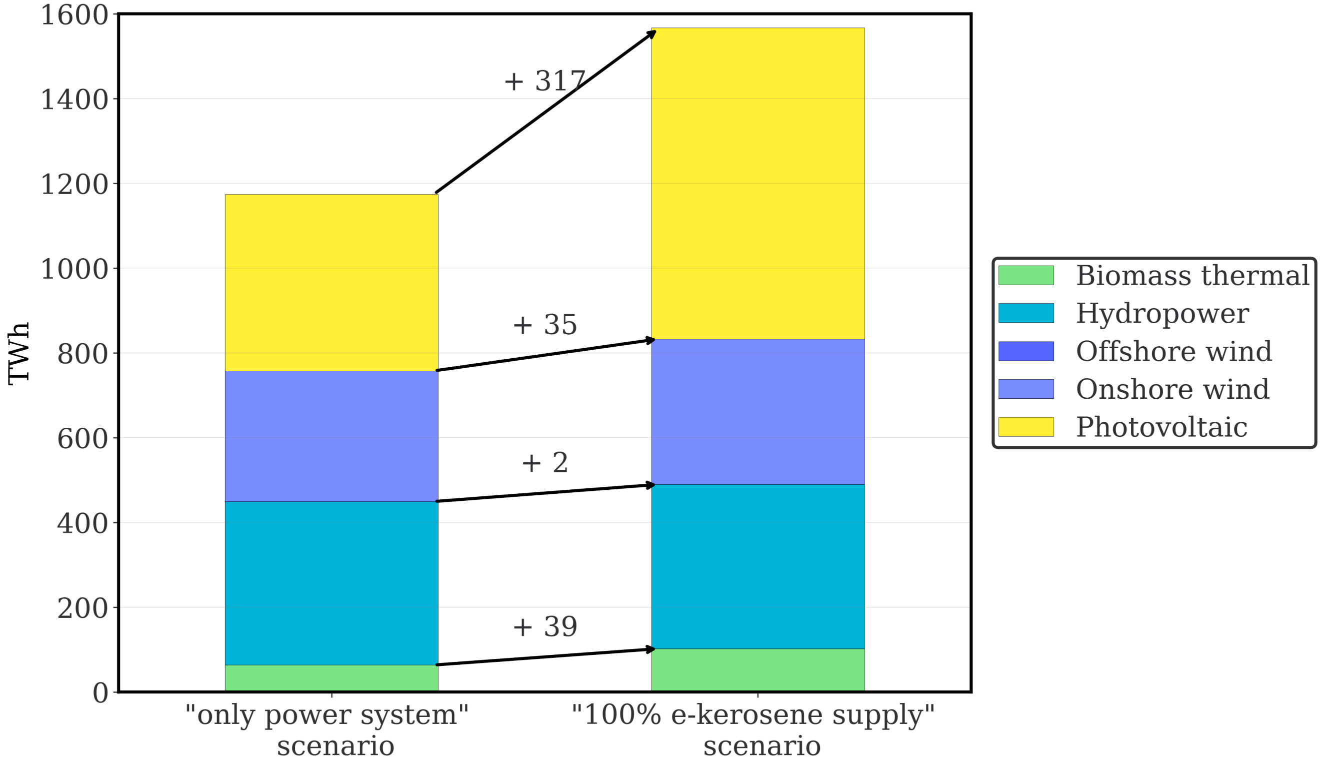

Figure S9. A comparison of annual electricity generation in Brazil's energy system under carbon-neutral scenarios without ("only power system") and with the integration of e-kerosene production ("100% e-kerosene supply").

Table S7. Gross Domestic Product (GDP), population, area, and population density in 2018.

Federal state	GDP (thousandR$)[S76]	Population[S77]	Area (km^2)[S78]	Population density (people/km^2)
São Paulo	2,210,561,949	45,538,936	248,219	184
Goiás	195,681,724	6,921,161	340,125	20
Distrito Federal	254,817,204	2,974,703	5760	516
Minas Gerais	614,875,819	21,040,662	586,521	36
Rio de Janeiro	758,859,046	17,159,960	43,750	392

E.2.2 A look into e-kerosene's mild contribution

The economic practicability of e-kerosene supply, as portrayed in Main Text Table 3, might lead one to expect its extensive incorporation in the fuel mix. However, an unexpected observation emerges from Main Text Figure 2, which reveals that even amidst high carbon pricing and biokerosene cost, e-kerosene accounts for a maximum of 51.1% of the total supply in the scenarios studied. For clarification, Figure S10 illustrates the allocation of biokerosene and e-kerosene across federal states, considering high biokerosene costs and carbon prices. The data in Figure S10 indicate that, in the majority of federal states, e-kerosene assumes the larger portion of the fuel supply. São Paulo, however, deviates from this trend, exhibiting a marked inclination for biokerosene, thus magnifying its cumulative share in fulfilling the kerosene demand.

A deeper examination of costs in São Paulo sheds light on prominence of biokerosene. Within PyPSA-Brazil, the biokerosene production cost in São Paulo is assumed to be 184.8 €/MWh, lower than the e-kerosene LCOF (about 215.4 €/MWh). An essential point of note is that the production cost of biokerosene is considered constant, whereas the cost of e-kerosene varies over time as calculated by PyPSA-Brazil. Consequently, e-kerosene's contribution is optimised during periods wherein its supply proves more economically efficient compared to biokerosene, which explains its modest share.

São Paulo's reliance on biokerosene has an overall impact on the fuel landscape in Brazil. The economic barriers that e-kerosene faces in competing with biokerosene are illustrated by the entrenched dominance of biokerosene in São Paulo, the state with the highest kerosene demand. To strengthen the market position of e-kerosene, efforts should be made to reduce its

production costs or policy reforms should be made to create an enabling environment for e-kerosene.

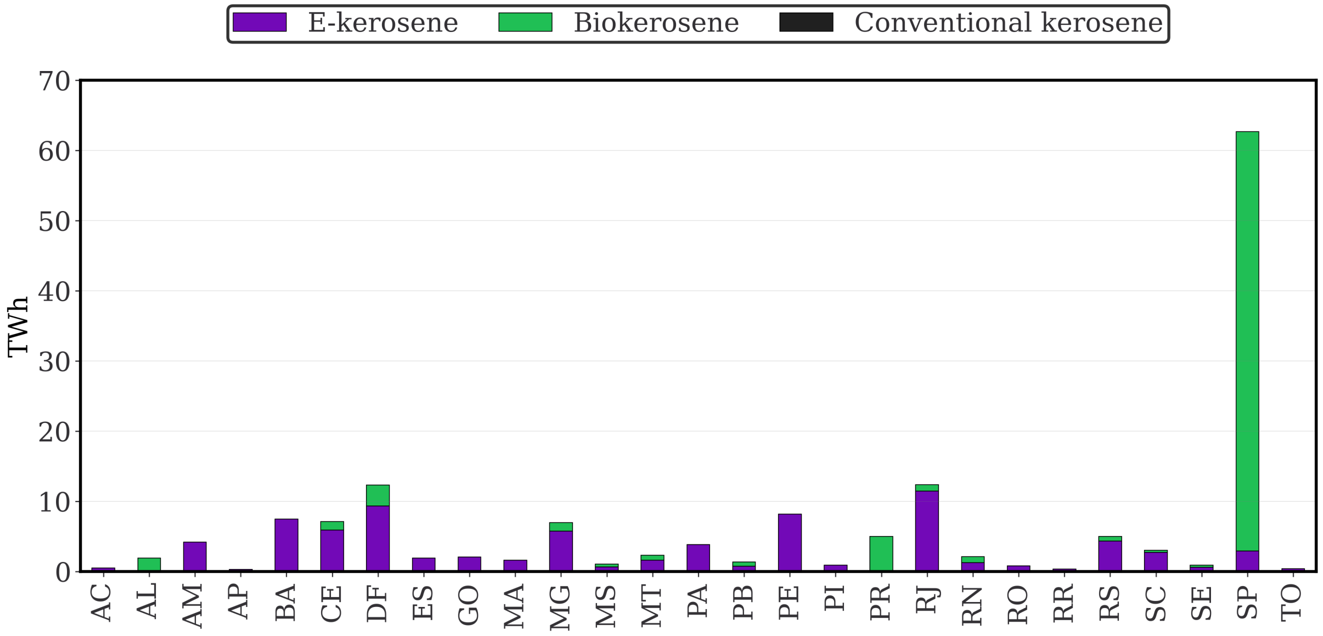

Figure S10. Supply of kerosene at federal state given the carbon price of 1000 €/t and biokerosene production level at high.

E.2.3 Biokerosene's impact on Brazil's role as an exporter of carbon-neutral kerosene

Main Text *Section Export costs of carbon-neutral kerosene from Brazil* explores Brazil's potential to become an exporter of carbon-neutral kerosene. As exhibited in Main Text Figure 3, the contribution of e-kerosene appears relatively modest when biokerosene production costs are low to medium. In those scenarios, biokerosene remains the primary export due to its affordability and presumed carbon-neutrality in PyPSA-Brazil. However, as the costs of biokerosene production increase, e-kerosene becomes more noticeable. A corresponding marked influence of biokerosene costs on the export costs and e-kerosene's export prominence is observable in Main Text *Section Export costs of carbon-neutral kerosene from Brazil*.

Assessing the likelihood of biokerosene production becoming high-cost within Brazil's geographic context is intricate due to various dynamic factors[S79]. However, biokerosene production demands substantial water and land resources, which could contribute to cost augmentations in the future[S44].

Additionally, the environmental sustainability of biokerosene is called into question by the risk of indirect GHG emissions arising from land-use changes. For instance, the HEFA pathway using cooking oil as feedstock, despite being cost-effective[S23], may emit as much as 27-67.4 $g\,CO_2/MJ$[S80,S81]. Although such levels of emissions are compliant with the sustainability criteria for SAF – which aim to reduce life-cycle GHG emissions by 10% compared to conventional kerosene[S82] – they cast doubts on the complete climate neutrality of biokerosene. The imperative to restrain biomass cultivation to safeguard biodiversity[S79], mitigate indirect GHG emissions[S69], and ensure responsible land use[S79] makes the presumption of limitless biokerosene production improbable. These factors could subsequently lead to a more conservative deployment of biokerosene at scale and, conversely, higher cost levels.

In light of these considerations, the ambition for Brazil to establish itself as an exporter of carbon-neutral kerosene may act as a catalyst for heightened investments in e-kerosene production. This would signify a progression from mere pursuit of a self-sufficiency in kerosene supply in 2050 to engaging proactively in global kerosene markets towards carbon-neutral aviation. The implications of such a transition could extend beyond economic considerations to involve broader sustainability objectives[S83]. Harnessing the potential of e-kerosene could position Brazil as a leader in the transition towards carbon-neutral kerosene, while concurrently supporting its commitments to climate change mitigation. This strategic approach would enable Brazil to strike a balance between economic, environmental, and societal interests, contributing significantly to the global endeavour of creating a sustainable aviation sector[S83].

E.2.4 Comparison to related literature

Electricity supply cost in carbon-neutral scenarios This section evaluate carbon-neutral Brazilian energy systems from our study and contrasts them with alternative designs presented in existing literature, focusing on electricity supply cost, represented by ASC (cf. Equation (15)).

The results from the "only power system" scenario in our study indicate an ASC of 50.3 €/MWh. This is lower than the 70.6 $€_{2019}$/MWh posited by Dranka and Ferreira[S9] for a 100% renewable power system across four Brazilian regions in 2050. The 70.6 €/MWh is converted from the original 78.99 $/MWh using 2019 exchange rates as indicated in Main Text Table 2. The discrepancy in ASC between our study and Dranka and Ferreira[S9] can be explained by differences in system designs. First, Dranka and Ferreira[S9] assume a higher rate of electrification, resulting in an electricity demand of 1,571.5 TWh, while we estimate a more conservative demand of 1,164.8 TWh ($COPPE_{lowBECCS}$ scenario presented in Main Text Table 2). Secondly, Dranka and Ferreira[S9] suggest a total power generation capacity of 623 GW, much larger than the 523 GW identified in our study. Furthermore, their study estimates hydropower to contribute 50% of energy supply, approximately 800 TWh, which is almost twice as much as Brazil's hydropower production capacity in 2019. In comparison, our study suggests a smaller share of 32.8% for hydropower (386-388 TWh, akin to the levels in 2019, cf. Main Text Table 1) and a higher share of 35.5% for PV. The capital costs and lifetime assumptions for PV also differ. Dranka and Ferreira[S9] lean less on solar power due to a permissive technology and economic parameterisation – their annualised capital costs of 187,357.6 €/MWh, nearly three times higher than the 48,931.5 €/MWh observed in our study.

Comparisons can also be made with Barbosa et al.[S11], which examines potential fully decarbonised Brazilian power systems with applications of renewable electricity for other end use. They report a decrease in ASC from 61 €/MWh to 53 €/MWh when adding 25% more electricity demand for water desalination and synthetic gas production. In a similar vein, we include e-kerosene production, which leads to a 32% increase in additional electricity demand under the "100% e-kerosene supply" scenario. Consequently, our ASC of the system dips from 50.3 €/MWh to 44.2 €/MWh. One of the key factors for the lower ASC in our study is the inclusion of interstate transmission in the PyPSA-Brazil performed, in contrast to the coarser spatial resolution in four regions adopted by Barbosa et al.[S11]. Furthermore, while Barbosa et al.[S11] include pumped and run-of-river hydropower plants and allows hydropower capacity to expand significantly – 200% of the installed capacity, we assume reservoir types with limited expansion capabilities, in line with Brazil's National Ten-Year Energy Plan[S19]. This assumption, although seemingly conservative, is based on Brazil's objective to diversify its energy generation portfolio, mitigating risks associated with dependency on hydropower, especially considering the environmental sensitivities in the Amazon region[S6].

In summary, our study unveils carbon-neutral power system designs for Brazil, which largely concur with those found in the existing literature. Notably, it employs the high-granularity PyPSA-Brazil model, integrating interstate transmission benefits even in the face of hydropower expansion constraints. Through this approach, a refined analysis is enabled, capitalising on Brazil's geographically diverse renewable resources. Consequently, our study presents a more cost-effective system than previously estimated in alternative literature, reducing the average system cost and affirming carbon neutrality as an attainable and economically viable goal for Brazil's power sector, whilst incorporating e-kerosene production.

Capacity factor of e-kerosene production unit Apart from securing a low electricity supply cost, a high capacity factor for the e-kerosene production unit is indispensable to ensure its economic feasibility[S84]. Echoing this perspective, Agora Energiewende[S85] present a comparison between scenarios with varying operation hours of electrolysers and synthesis, specifically 2,000 and 8,000 hours. In addition, Breyer et al.[S34] highlight the considerable impact that the operation hours of FT synthesis have on economic viability as they assume baseload-like DAC operating for 8,000 hours. Our study aligns with these findings, demonstrating that the capacity factor for e-kerosene production fluctuates between 0.7 and 0.9 – a range that supports its economic feasibility.

Levelised cost of fuel for e-kerosene supply

Table S8. LCOF for e-kerosene in 2050 as reported in the literature.

Study	Country	LCOF[a] ($€_{2019}$/MWh)	Notable factors influencing cost
Our study[b]	Brazil	214.7	Fully decarbonised power system with e-kerosene meeting 100% of the kerosene demand ("100% e-kerosene supply" scenario).
Our study	Brazil	113.3 – 215.5	Scenarios excluding exports, considering variable biokerosene production costs (low, medium, and high) and carbon prices (160 €/t, 200 €/t, 260 €/t, 320 €/t, 500 €/t, 1000 €/t).
Our study	Brazil	117.9 – 227.3	Scenarios including various export demand levels (50%, 100%, 150%, 200%, 400%, and 500%) with fixed high carbon prices at 1000 €/t, considering biokerosene production costs (low, medium, and high) and exclusive e-kerosene.
S84	Germany	186.9	The value, initially 2186 €/t, is based on electricity supply cost at 43 €/MWh [S84, Table 13], employing the identical e-kerosene plant setup as the dissertation.

Continued on next page

Study	Country	LCOF[a] (€$_{2019}$/MWh)	Comment
S32	Germany	244.8 – 284.3	The value ranges from 2.91 €/kg to 3.38 €/kg. Power-to-liquid process with low-temperature electrolysis, CO_2 from ambient air and renewable electricity cost varying from 35 €/MWh to 55 €/MWh [S32, Table 7]. This cost is the cost of electricity used to meet a specific e-kerosene demand, driven by the specific process, process efficiency and carrier requirements (e.g. CO_2).
S32	Germany	188.4 – 227.1	This range, originally 2.24 €/kg to 2.7 €/kg) [S32, Table 7]. Similar to the previous entry but using high-temperature electrolysis.
S86	Spain	188.4 - 416.4	This range [S86, Figure 7] relies on the electricity source (renewable or grid) and the incorporation of emissions costs. Utilising renewable electricity, the LCOF is estimated at 2.3 €/kg without emission cost and increase to 4.95 €/kg when considering the emission costs. Grid-sourced electricity results in a smaller increment due to emissions costs (from 2.24 €/kg to 2.8 €/kg). The study utilises a MILP approach to optimise the total system cost of the e-kerosene supply chain at 36 nodes.
S84	Spain	148.2	Based on FT synthesis with DAC and electricity supply cost at 35 €/MWh, the original value is 1762 €/t [S84, Table 13].
S84	Morocco	145.9	Similar to Spain but with electricity supply cost at 32 €/MWh. The original value was 1735 €/t [S84, Table 13].
S85	North Africa	75 – 137.5	The range, 75 €/MWh to 137.5 €/MWh [S85, Figure 21, 2050, 100%], indicates the site of liquid synthetic fuel production and import to Germany, which includes transport costs. Note that e-kerosene is not explicitly mentioned as the final product.
S34	EU-27	75	In this study, FT-derived kerosene is produced using DAC with electricity sourced from hourly PV and wind energy generation. Electricity costs are calculated without grid expenses. The valued is from [S34, Table 11].
S34	US	69	The assumptions mirror the EU-27 scenario, but this scenario is conducted for the US, which has a different renewable energy generation potential.
S24	US[c]	84.8	The raw value is converted from 0.99 $/L_*gasoline equivalent* to $/MWh using density and heat value of gasoline of 0.755 kg/L and 13 kWh/kg) from [S24, page F]. The study employs grid electricity at 65 $/MWh,[d] wind supply at 19 $/MWh, and solar supply at 11.8 $/MWh.[e]
S87	—	217.0 – 434.1	The range (2-4 €/L) in the study represents a scenario in 2050 where CO_2 from DAC and H_2 from water electrolysis are combined to produce synthetic fuels using renewable electricity, supplied based on LCOE at 61 €/MWh [S87, Figure 11, 2050 DAC-CCU scenario].

[a] Unit conversion details are elaborated in Table S5.
[b] The formulation details can be found in Equation (17).
[c] The data on supply costs of electricity originate from the US.
[d] Sherwin[S24] state that grid power not necessary for green power generation.
[e] The data are derived from the supplementary materials.

References of supplementary material

S1. Chang, M. *et al.* Trends in tools and approaches for modelling the energy transition. *Appl. Energy* **290**, 116731, DOI: 10.1016/j.apenergy.2021.116731 (2021).

S2. Prina, M. G., Manzolini, G., Moser, D., Nastasi, B. & Sparber, W. Classification and challenges of bottom-up energy system models - A review. *Renew. Sustain. Energy Rev.* **129**, 109917, DOI: 10.1016/j.rser.2020.109917 (2020).

S3. Ringkjøb, H.-K., Haugan, P. M. & Solbrekke, I. M. A review of modelling tools for energy and electricity systems with large shares of variable renewables. *Renew. Sustain. Energy Rev.* **96**, 440–459, DOI: 10.1016/j.rser.2018.08.002 (2018).

S4. Poncelet, K., Delarue, E., Six, D., Duerinck, J. & D'haeseleer, W. Impact of the level of temporal and operational detail in energy-system planning models. *Appl. Energy* **162**, 631–643, DOI: 10.1016/j.apenergy.2015.10.100 (2016).

S5. Pfenninger, S., Hawkes, A. & Keirstead, J. Energy systems modeling for twenty-first century energy challenges. *Renew. Sustain. Energy Rev.* **33**, 74–86, DOI: 10.1016/j.rser.2014.02.003 (2014).

S6. Nogueira, L. P. P. *et al.* Will thermal power plants with CCS play a role in Brazil's future electric power generation? *Int. J. Greenh. Gas Control.* **24**, 115–123, DOI: 10.1016/j.ijggc.2014.03.002 (2014).

S7. Rochedo, P. R. R. *et al.* The threat of political bargaining to climate mitigation in Brazil. *Nat. Clim. Chang.* **8**, 695–698, DOI: 10.1038/s41558-018-0213-y (2018).

S8. Fichter, T., Soria, R., Szklo, A., Schaeffer, R. & Lucena, A. F. Assessing the potential role of concentrated solar power (CSP) for the northeast power system of Brazil using a detailed power system model. *Energy* **121**, 695–715, DOI: 10.1016/j.energy.2017.01.012 (2017).

S9. Dranka, G. G. & Ferreira, P. Planning for a renewable future in the Brazilian power system. *Energy* **164**, 496–511, DOI: 10.1016/j.energy.2018.08.164 (2018).

S10. Gils, H., Simon, S. & Soria, R. 100% Renewable Energy Supply for Brazil—The Role of Sector Coupling and Regional Development. *Energies* **10**, 1859, DOI: 10.3390/en10111859 (2017).

S11. Barbosa, L. d. S. N. S., Orozco, J. F., Bogdanov, D., Vainikka, P. & Breyer, C. Hydropower and Power-to-gas Storage Options: The Brazilian Energy System Case. *Energy Procedia* **99**, 89–107, DOI: 10.1016/j.egypro.2016.10.101 (2016).

S12. Kriechbaum, L., Scheiber, G. & Kienberger, T. Grid-based multi-energy systems—modelling, assessment, open source modelling frameworks and challenges. *Energy, Sustain. Soc.* **8**, 35, DOI: 10.1186/s13705-018-0176-x (2018).

S13. Pfenninger, S. *et al.* Opening the black box of energy modelling: Strategies and lessons learned. *Energy Strateg. Rev.* **19**, 63–71, DOI: 10.1016/j.esr.2017.12.002 (2018).

S14. Müller-Casseres, E. *et al.* Production of alternative marine fuels in Brazil: An integrated assessment perspective. *Energy* **219**, 119444, DOI: 10.1016/j.energy.2020.119444 (2021).

S15. Brown, T., Hörsch, J. & Schlachtberger, D. PyPSA: Python for Power System Analysis. *J. Open Res. Softw.* **6**, 4, DOI: 10.5334/jors.188 (2018).

S16. Hörsch, J., Hofmann, F., Schlachtberger, D. & Brown, T. PyPSA-Eur: An Open Optimisation Model of the European Transmission System. *Energy Strateg. Rev.* **22**, 207–215, DOI: 10.1016/j.esr.2018.08.012 (2018).

S17. Short, W., Packey, D. & Holt, T. A manual for the economic evaluation of energy efficiency and renewable energy technologies. Tech. Rep. NREL/TP–462-5173, 35391, National Renewable Energy Lab (NREL) (1995). DOI: 10.2172/35391. http://www.osti.gov/servlets/purl/35391-NqycFd/webviewable/.

S18. Schlachtberger, D., Brown, T., Schramm, S. & Greiner, M. The benefits of cooperation in a highly renewable European electricity network. *Energy* **134**, 469–481, DOI: 10.1016/j.energy.2017.06.004 (2017).

S19. Deng, Y. *et al.* Harmonized and Open Energy Dataset for Modeling a Highly Renewable Brazilian Power System. *Sci. Data* **10**, 103, DOI: 10.1038/s41597-023-01992-9 (2023).

S20. Hörsch, J., Ronellenfitsch, H., Witthaut, D. & Brown, T. Linear optimal power flow using cycle flows. *Electr. Power Syst. Res.* **158**, 126–135, DOI: 10.1016/j.epsr.2017.12.034 (2018).

S21. Moseley, P. T. & Garche, J. *Electrochemical Energy Storage for Renewable Sources and Grid Balancing* (Elsevier, 2015).

S22. EPE. Plano Decenal de Expansão de Energia 2029. Tech. Rep., Empresa de Pesquisa Energética (EPE) (2020). https://www.epe.gov.br/sites-pt/publicacoes-dados-abertos/publicacoes/Documents/PDE%202029.pdf.

S23. Schmidt, P. & Weindorf, W. Power-to-Liquids: Potentials and Perspectives. Tech. Rep., Ludwig-Bölkow-Systemtechnik GmbH (LBST) (2016). https://www.umweltbundesamt.de/sites/default/files/medien/377/publikationen/161005_uba_hintergrund_ptl_barrierrefrei.pdf.

S24. Sherwin, E. D. Electrofuel Synthesis from Variable Renewable Electricity: An Optimization-Based Techno-Economic Analysis. *Environ. Sci. & Technol.* **55**, 7583–7594, DOI: 10.1021/acs.est.0c07955 (2021).

S25. Schmidt, P., Zittel, W., Weindorf, W., Rakasha, T. & Goericke, D. Renewables in transport 2050 – Empowering a sustainable mobility future with zero emission fuels. In Bargende, M., Reuss, H.-C. & Wiedemann, J. (eds.) *16. Internationales Stuttgarter Symposium*, Proceedings, 185–199, DOI: 10.1007/978-3-658-13255-2_15 (Springer Fachmedien, Wiesbaden, 2016).

S26. Ueckerdt, F. *et al.* Potential and risks of hydrogen-based e-fuels in climate change mitigation. *Nat. Clim. Chang.* **11**, 384–393, DOI: 10.1038/s41558-021-01032-7 (2021).

S27. Gabrielli, P., Gazzani, M. & Mazzotti, M. The Role of Carbon Capture and Utilization, Carbon Capture and Storage, and Biomass to Enable a Net-Zero-CO2 Emissions Chemical Industry. *Ind. & Eng. Chem. Res.* **59**, 7033–7045, DOI: 10.1021/acs.iecr.9b06579 (2020).

S28. Gleeson, T., Befus, K. M., Jasechko, S., Luijendijk, E. & Cardenas, M. B. The global volume and distribution of modern groundwater. *Nat. Geosci.* **9**, 161–167, DOI: 10.1038/ngeo2590 (2016).

S29. Pekel, J.-F., Cottam, A., Gorelick, N. & Belward, A. S. High-resolution mapping of global surface water and its long-term changes. *Nature* **540**, 418–422, DOI: 10.1038/nature20584 (2016).

S30. Stockli, R. Sea Surface Temperature, Salinity and Density, URL https://svs.gsfc.nasa.gov/3652 (2009).

S31. Alves, K. d. F. *et al.* Water scarcity in Brazil: part 2—uncertainty assessment in regionalized characterization factors. *The Int. J. Life Cycle Assess.* **25**, 2359–2379, DOI: 10.1007/s11367-020-01739-3 (2020).

S32. Drünert, S., Neuling, U., Zitscher, T. & Kaltschmitt, M. Power-to-Liquid fuels for aviation – Processes, resources and supply potential under German conditions. *Appl. Energy* **277**, 115578, DOI: 10.1016/j.apenergy.2020.115578 (2020).

S33. ASTM Committee D02. D7566-18: Specification for Aviation Turbine Fuel Containing Synthesized Hydrocarbons. Tech. Rep., ASTM International (2018). DOI: 10.1520/D7566-18. http://www.astm.org/cgi-bin/resolver.cgi?D7566-18.

S34. Breyer, C. *et al.* E-kerosene for Commercial Aviation: From Green Hydrogen and CO2 from Direct Air Capture – Volumes, Cost, Area Demand and Renewable Energy Competition in the United States and Europe from 2030 to 2050. Tech. Rep., Deutsche Energie-Agentur (dena) (2022). https://www.dena.de/fileadmin/dena/Publikationen/PDFs/2022/STUDY_E-Kerosene_for_Commercial_Aviation.pdf.

S35. ATAG. Waypoint 2050: An Air Transport Action Group Project. Tech. Rep., ATAG, Geneva, Switzerland, (2021). https://aviationbenefits.org/media/167417/w2050_v2021_27sept_full.pdf.

S36. Micheli, M., Moore, D., Bach, V. & Finkbeiner, M. Life-Cycle Assessment of Power-to-Liquid Kerosene Produced from Renewable Electricity and CO2 from Direct Air Capture in Germany. *Sustainability* **14**, 10658, DOI: 10.3390/su141710658 (2022).

S37. Stratton, R. W., Wolfe, P. J. & Hileman, J. I. Impact of Aviation Non-CO_2 Combustion Effects on the Environmental Feasibility of Alternative Jet Fuels. *Environ. Sci. & Technol.* **45**, 10736–10743, DOI: 10.1021/es2017522 (2011).

S38. Braun-Unkhoff, M., Riedel, U. & Wahl, C. About the emissions of alternative jet fuels. *CEAS Aeronaut. J.* **8**, 167–180, DOI: 10.1007/s13272-016-0230-3 (2017).

S39. König, D. H., Freiberg, M., Dietrich, R.-U. & Wörner, A. Techno-economic study of the storage of fluctuating renewable energy in liquid hydrocarbons. *Fuel* **159**, 289–297, DOI: 10.1016/j.fuel.2015.06.085 (2015).

S40. ONS. EAR Diário por Subsistema: 2019, URL https://dados.ons.org.br/dataset/ear-diario-por-subsistema (2022).

S41. ANEEL. Resultado do Leilão de Transmissão, URL https://www.aneel.gov.br/documents/654791/20589082/Resultado+do+Leil%C3%A3o+de+Transmiss%C3%A3o/0ac53811-f890-9274-1ba9-4bee7cab9723 (2021).

S42. EPE & MME. Plano Nacional de Energia 2050. Tech. Rep., Ministério de Minas e Energia (MME), Empresa de Pesquisa Energética (EPE), Brasília, Brazil (2020). https://www.epe.gov.br/pt/publicacoes-dados-abertos/publicacoes/Plano-Nacional-de-Energia-2050.

S43. Schröder, A., Kunz, F., Meiss, J. & Mendelevitch, R. Current and prospective costs of electricity generation until 2050. Tech. Rep. DIW Data Documentation No. 68, Deutsches Institut für Wirtschaftsforschung (DIW), Berlin (2013). https://www.econstor.eu/handle/10419/80348.

S44. Schmidt, P., Batteiger, V., Roth, A., Weindorf, W. & Raksha, T. Power-to-Liquids as Renewable Fuel Option for Aviation: A Review. *Chemie Ingenieur Tech.* **90**, 127–140, DOI: 10.1002/cite.201700129 (2018).

S45. Fuel Tank Shop. 10000 Litre Bunded Steel Oil Tank
, URL https://www.fueltankshop.co.uk/10000-litre-bunded-steel-oil-tank/p4156 (2023).

S46. European Central Bank. Euro reference exchange rate: Pound sterling (GBP), URL https://www.ecb.europa.eu/stats/policy_and_exchange_rates/euro_reference_exchange_rates/html/eurofxref-graph-gbp.en.html (2023).

S47. EPA. GHG Emission Factors Hub, URL https://www.epa.gov/climateleadership/ghg-emission-factors-hub (2022).

S48. European Central Bank. Euro reference exchange rate: US dollar (USD), URL https://www.ecb.europa.eu/stats/policy_and_exchange_rates/euro_reference_exchange_rates/html/eurofxref-graph-usd.en.html (2022).

S49. European Central Bank. Euro reference exchange rate: Brazilian real (BRL), URL https://www.ecb.europa.eu/stats/policy_and_exchange_rates/euro_reference_exchange_rates/html/eurofxref-graph-brl.en.html (2022).

S50. Newell, D. B. & Tiesinga, E. *The international system of units (SI): 2008 edition*. NIST SP 330, 2008 edition (National Institute of Standards and Technology, Gaithersburg, MD, 2019).

S51. DEA. Technology Data for Generation of Electricity and District Heating, URL https://ens.dk/en/our-services/projections-and-models/technology-data/technology-data-generation-electricity-and (2020).

S52. ONS. Reserva Girante na Demanda Máxima do SIN, URL https://sdro.ons.org.br/SDRO/DIARIO/2021_06_28/HTML/13_ReservaGiranteDemandaMaxima.html (2021).

S53. Bogdanov, D. *et al.* Radical transformation pathway towards sustainable electricity via evolutionary steps. *Nat. Commun.* **10**, 1077, DOI: 10.1038/s41467-019-08855-1 (2019).

S54. IEA. World Energy Outlook 2021. License: CC BY 4.0, International Energy Agency (IEA), IEA, Paris (2021). https://www.iea.org/reports/world-energy-outlook-2021.

S55. Hagspiel, S. *et al.* Cost-optimal power system extension under flow-based market coupling. *Energy* **66**, 654–666, DOI: 10.1016/j.energy.2014.01.025 (2014).

S56. DEA. Technology Data for Energy Storage, URL https://ens.dk/en/our-services/projections-and-models/technology-data/technology-data-energy-storage (2020).

S57. DEA. Technology Data for Renewable Fuels, URL https://ens.dk/en/our-services/projections-and-models/technology-data/technology-data-renewable-fuels (2021).

S58. Maurice, S. *Surface Production Operations: Volume 5: Pressure Vessels, Heat Exchangers, and Aboveground Storage Tanks* (Gulf Professional Publishing, 2021), 1st edition edn.

S59. Moriarty, K. & Kvien, A. U.S. Airport Infrastructure and Sustainable Aviation Fuel. Tech. Rep. NREL/TP–5400-78368, 1768316, MainId:32285, Federal Aviation Administration (FAA) (2021). DOI: 10.2172/1768316. https://www.osti.gov/servlets/purl/1768316/.

S60. ANAC. Dados Estatísticos do Transporte Aéreo no Brasil: Microdados, URL https://www.gov.br/anac/pt-br/assuntos/regulados/empresas-aereas/Instrucoes-para-a-elaboracao-e-apresentacao-das-demonstracoes-contabeis/envio-de-informacoes/microdados (2021).

S61. Ministry of Infrastructure. Brazil's Action Plan on CO2 Emissions Reduction from Aviation: Base Year 2018. Govermental, Ministry of Infrastructure (2019). https://www.gov.br/anac/pt-br/assuntos/meio-ambiente/arquivos/BrazilsActionPlanonCO2EmissionsReductionfromAviation3rdEditionBaseYear2018.pdf.

S62. ICAO. Guidance on the Development of States' Action Plan on CO2 Emissions Reduction Activities (Doc 9988), URL https://store.icao.int/en/guidance-on-the-development-of-states-action-plan-on-co2-emissions-reduction-activities-doc-9988 (2019).

S63. ANP. Preços de distribuição de combustíveis: Combustíveis de aviação, Mensais, URL https://www.gov.br/anp/pt-br/assuntos/precos-e-defesa-da-concorrencia/precos/precos-de-distribuicao-de-combustiveis (2022).

S64. Cervi, W. R. *et al.* Spatial modeling of techno-economic potential of biojet fuel production in Brazil. *GCB Bioenergy* **12**, 136–157, DOI: 10.1111/gcbb.12659 (2020).

S65. Prussi, M. *et al.* CORSIA: The first internationally adopted approach to calculate life-cycle GHG emissions for aviation fuels. *Renew. Sustain. Energy Rev.* **150**, 111398, DOI: 10.1016/j.rser.2021.111398 (2021).

S66. UNFCCC. Federative Republic of Brazil: Paris Agreement: Nationally determined contribution (NDC). Tech. Rep., United Nations Framework Convention on Climate Change (UNFCCC), Brasília, Brazil (2022). https://unfccc.int/sites/default/files/NDC/2022-06/Updated%20-%20First%20NDC%20-%20%20FINAL%20-%20PDF.pdf.

S67. EPE. Anuário estatístico de energia elétrica 2011, URL https://www.epe.gov.br/sites-pt/publicacoes-dados-abertos/publicacoes/PublicacoesArquivos/publicacao-160/topico-168/Anu%C3%A1rio%20Estat%C3%ADstico%20de%20Energia%20El%C3%A9trica%202011.pdf (2011).

S68. ICAO. Resolution A40-18: Consolidated statement of continuing ICAO policies and practices related to environmental protection - Climate change. Tech. Rep., International Civil Aviation Organization (ICAO) (2019). https://www.icao.int/environmental-protection/Documents/Assembly/Resolution_A40-18_Climate_Change.pdf.

S69. World Bank. State and Trends of Carbon Pricing 2022. Serial, World Bank, Washington, DC (2022). https://openknowledge.worldbank.org/handle/10986/37455.

S70. Lee, D. S. *et al.* The contribution of global aviation to anthropogenic climate forcing for 2000 to 2018. *Atmospheric Environ.* **244**, 117834, DOI: 10.1016/j.atmosenv.2020.117834 (2021).

S71. European Commission. Updated analysis of the non-CO2 climate impacts of aviation and potential policy measures pursuant to EU Emissions Trading System Directive Article 30(4). Tech. Rep., European Commission (2020). https://eur-lex.europa.eu/legal-content/EN/TXT/PDF/?uri=CELEX:52020DC0747.

S72. IPCC. Mitigation Pathways Compatible with 1.5°C in the Context of Sustainable Development. In *Global Warming of 1.5°C. An IPCC Special Report on the impacts of global warming of 1.5°C above pre-industrial levels and related global greenhouse gas emission pathways, in the context of strengthening the global response to the threat of climate change, sustainable development, and efforts to eradicate poverty*, 82, DOI: 10.1017/9781009157940 (Cambridge University Press, 2022), 1 edn.

S73. Brown, T. & Reichenberg, L. Decreasing market value of variable renewables can be avoided by policy action. *Energy Econ.* **100**, 105354, DOI: 10.1016/j.eneco.2021.105354 (2021).

S74. Fitiwi, D. Z., Lynch, M. & Bertsch, V. Power system impacts of community acceptance policies for renewable energy deployment under storage cost uncertainty. *Renew. Energy* **156**, 893–912, DOI: 10.1016/j.renene.2020.03.110 (2020).

S75. Bertsch, V., Hall, M., Weinhardt, C. & Fichtner, W. Public acceptance and preferences related to renewable energy and grid expansion policy: Empirical insights for Germany. *Energy* **114**, 465–477, DOI: 10.1016/j.energy.2016.08.022 (2016).

S76. IBGE. Produto Interno Bruto dos Municípios, URL https://www.ibge.gov.br/estatisticas/economicas/contas-nacionais/9088-produto-interno-bruto-dos-municipios.html?edicao=29720&t=downloads (2018).

S77. IBGE. Estimativas da população residente para os municípios e para as unidades da federação | IBGE, URL https://www.ibge.gov.br/estatisticas/sociais/populacao/9103-estimativas-de-populacao.html?=&t=downloads (2018).

S78. IBGE. Áreas Territoriais, URL https://www.ibge.gov.br/geociencias/organizacao-do-territorio/estrutura-territorial/15761-areas-dos-municipios.html?edicao=24050&t=acesso-ao-produto (2018).

S79. Kaltschmitt, M. & Neuling, U. (eds.) *Biokerosene: Status and Prospects* (Springer Berlin Heidelberg, Berlin, Heidelberg, 2018).

S80. De Jong, S. *et al.* Life-cycle analysis of greenhouse gas emissions from renewable jet fuel production. *Biotechnol. for Biofuels* **10**, 64, DOI: 10.1186/s13068-017-0739-7 (2017).

S81. ICAO. CORSIA Default Life Cycle Emissions Values for CORSIA Eligible Fuels. Tech. Rep., ICAO (2021). https://www.icao.int/environmental-protection/CORSIA/Documents/ICAO%20document%2006%20-%20Default%20Life%20Cycle%20Emissions%20-%20March%202021.pdf.

S82. ICAO. CORSIA Sustainability Criteria for CORSIA Eligible Fuels. Tech. Rep., ICAO (2021). https://www.icao.int/environmental-protection/CORSIA/Documents/ICAO%20document%2005%20-%20Sustainability%20Criteria%20-%20November%202021.pdf.

S83. Bergero, C. *et al.* Pathways to net-zero emissions from aviation. *Nat. Sustain.* DOI: 10.1038/s41893-022-01046-9 (2023).

S84. Batteiger, V. *et al.* Power-to-Liquids – A scalable and sustainable fuel supply perspective for aviation. Tech. Rep., German Environment Agency (2022). https://www.umweltbundesamt.de/en/publikationen/power-to-liquids.

S85. Agora Energiewende. The Future Cost of Electricity-Based Synthetic Fuels. Tech. Rep., Agora Energiewende, Berlin, Germany (2018). https://www.agora-energiewende.de/en/publications/the-future-cost-of-electricity-based-synthetic-fuels-1/.

S86. Gonzalez-Garay, A. *et al.* Unravelling the potential of sustainable aviation fuels to decarbonise the aviation sector. *Energy & Environ. Sci.* **15**, 3291–3309, DOI: 10.1039/D1EE03437E (2022).

S87. Becattini, V., Gabrielli, P. & Mazzotti, M. Role of Carbon Capture, Storage, and Utilization to Enable a Net-Zero-CO_2 -Emissions Aviation Sector. *Ind. & Eng. Chem. Res.* **60**, 6848–6862, DOI: 10.1021/acs.iecr.0c05392 (2021).
